# The Transcription Factor MAZR Preferentially Acts as a Transcriptional Repressor in Mast Cells and Plays a Minor Role in the Regulation of Effector Functions in Response to FcεRI Stimulation

**DOI:** 10.1371/journal.pone.0077677

**Published:** 2013-10-17

**Authors:** Anastasia Abramova, Shinya Sakaguchi, Alexandra Schebesta, Hammad Hassan, Nicole Boucheron, Peter Valent, Axel Roers, Wilfried Ellmeier

**Affiliations:** 1 Division of Immunobiology, Institute of Immunology, Center for Pathophysiology, Infectiology and Immunology, Medical University of Vienna, Vienna, Austria; 2 Division of Hematology and Hemostaseology, Department of Internal Medicine I, Medical University of Vienna, Vienna, Austria; 3 Institute for Immunology, University of Technology Dresden, Medical Faculty Carl-Gustav Carus, Dresden, Germany; Oklahoma Medical Research Foundation, United States of America

## Abstract

Mast cells are key players in type I hypersensitivity reactions in humans and mice and their activity has to be tightly controlled. Previous studies implicated the transcription factor MAZR in the regulation of mast cell function. To study the role of MAZR in mast cells, we generated a conditional *Mazr* allele and crossed *Mazr* ^F/F^ mice with the *Vav-iCre* deleter strain, which is active in all hematopoietic cells. MAZR-null BM-derived mast cells (BMMC) were phenotypically indistinguishable from wild-type BMMCs, although the numbers of IL-3 generated *Mazr* ^*F/F*^ *Vav-iCre* BMMCs were reduced in comparison to *Mazr* ^*F/F*^ BMMCs, showing that MAZR is required for the efficient generation of BMMC *in vitro*. A gene expression analysis revealed that MAZR-deficiency resulted in the dysregulation of 128 genes, with more genes up- than down-regulated in the absence of MAZR, indicating that MAZR acts as a transcriptional repressor in mast cells. Among the up-regulated genes were the chemokines *Ccl5*, *Cxcl10*, *Cxcl12*, the chemokine receptor *Ccr5* and the cytokine *IL18*, suggesting an immunoregulatory role for MAZR in mast cells. Enforced expression of MAZR in mature *Mazr*-deficient BMMCs rescued the altered expression pattern of some genes tested, suggesting direct regulation of these genes by MAZR. Upon FcεRI stimulation, *Mazr* expression was transiently down-regulated in BMMCs. However, early and late effector functions in response to FcεRI-mediated stimulation were not impaired in the absence of MAZR, with the exception of IL-6, which was slightly decreased. Taken together, out data indicate that MAZR preferentially acts as a transcriptional repressor in mast cells, however MAZR plays only a minor role in the transcriptional networks that regulate early and late effector functions in mast cells in response to FcεRI stimulation.

## Introduction

Mast cells are derived from hematopoietic progenitor cells that migrate to various tissues where they differentiate into tissue-resident mast cells [1]. Mast cells are known to be the key players in type I hypersensitivity reactions in humans and mice. They are critically involved in the development of allergic rhinitis, allergic asthma and systemic anaphylaxis. Mast cells express the high-affinity Fc receptor type I for Immunoglobulin (Ig) E (FcεRI) and thus are able to bind IgE. The classical activation of mast cells by crosslinking of the IgE/FcεRI complex with antigen (e.g. an allergen) induces a variety of early- and late-phase effector functions. During the early phase of mast cell activation that occurs within minutes, the cells secrete preformed mediators like histamine, proteolytic enzymes and proteoglycans. In addition, lipid mediators such as leukotrienes and prostaglandins are newly synthesized and released as a part of the early effector phase. Mast cell activation also leads to the production of various cytokines and chemokines, which characterizes the late-phase response of mast cell activation. Together, this large number of different mediators and factors produced during the early- and late-phase activation of mast cells is responsible for the many physiological and pathophysiological changes associated with type I hypersensitivity reactions [2]. Moreover, mast cells can also be activated by IgE/FcεRI-independent signals such as FcγR or complement receptors, by a variety of different Toll-like receptor (TLR) ligands, and they also play important roles in host defense against bacterial infections or toxins. In addition, mast cells have been implicated in the pathology of autoimmune diseases, inflammation and cancer [3-5]. Based on these important roles of mast cells, their activation has to be tightly controlled and thus it is important to understand signaling as well transcriptional networks that control their differentiation and activation.

 The transcription factor MAZR (also known as PATZ1, encoded by the *Patz1* gene, referred throughout the manuscript as *Mazr*) is a member of the BTB/POZ-domain containing family of Zn finger transcriptional regulators [6]. We recently identified that MAZR negatively regulates *Cd8* gene expression in DN thymocytes [7]. By generating *Mazr* ^*-/-*^ mice, we also showed that MAZR is a part of the transcription factor network that regulates CD4/CD8 cell fate choice [8]. Germline MAZR knockout mice on a mixed 129Sv/C57BL/6 background were born at severely reduced Mendelian ratio, were smaller in size than wild-type (wt) littermate controls and male MAZR-deficient mice were infertile due to defects in gametogenesis [8,9]. Moreover, MAZR-deficient mice backcrossed on a C57BL/6 background are embryonically lethal [8], most likely due to defects in the developing CNS and cardiac vessel anomalies [10]. MAZR has been implicated to function as a tumor suppressor gene [11]. Thus, MAZR has multiple roles inside and outside of the hematopoietic system.

 MAZR has also been linked with the regulation of mast cells. MAZR interacts with the mi transcription factor (MITF) [12], which is a basic-helix-loop-helix leucine zipper (bHLH-Zip) transcription factor that regulates a larger group of genes in mast cells involved in mast cell differentiation and survival [13-17]. It has been shown that MAZR is expressed in cultured mast cells from the spleen as well as in MST mastocytoma cells [12]. Overexpression of a putative dominant-negative version of MAZR consisting of aa 409–496 of the zinc-finger domain in MST mastocytoma cells led to reduced expression levels *Tpsb2* (encoding for Tryptase beta 2, also known as Mast cell protease 6), while overexpression of MAZR together with MITF in Jurkat T cells enhanced *Tpsb2* promoter activity [12]. These data suggest that MAZR might regulate gene expression in mast cells in association with MITF.

 To study the role of MAZR in primary mast cells, we generated a conditional *Mazr* allele and crossed *Mazr* ^F/F^ mice with the *Vav-iCre* deleter strain, which is active in all hematopoietic cells [18]. *Mazr* ^*F/F*^ *Vav-iCre* mice displayed a similar size as *Mazr* ^*F/F*^ mice, were fertile and born at normal Mendelian ratio, demonstrating that the loss of MAZR in hematopoietic cells is not the cause of the observed embryonic lethality in germline *Mazr* ^*-/-*^ mice. MAZR-deficient bone marrow-derived mast cells (BMMC) were phenotypically indistinguishable from wild-type BMMCs, although *Mazr* ^*F/F*^ *Vav-iCre* BM cultures yielded less BMMCs in comparison to *Mazr* ^*F/F*^ BM cultures, suggesting that MAZR is required for the efficient generation of BMMC *in vitro*. A gene expression analysis of MAZR-deficient BMMCs revealed that loss of MAZR altered the expression pattern of 128 genes and that more genes were up- than down-regulated in the absence of MAZR. This suggests that MAZR preferentially acts as a transcriptional repressor in mast cells. Among the genes that were up-regulated were cytokines (*Il18*), several chemokines (*Ccl5*, *Cxcl10*, *Cxcl12*) and the chemokine receptor *Ccr5*, suggesting an immunoregulatory role of MAZR in mast cells. Enforced expression of MAZR in mature *Mazr*-deficient BMMCs rescued the altered expression pattern of some genes that were tested, suggesting direct regulation of these genes by MAZR. Upon FcεRI stimulation, *Mazr* expression was transiently down-regulated during the first 3 hours of activation and it went back to the levels of non-stimulated cells after 24 hours. Although IL-6 production was slightly decreased in MAZR-null BMMCs, other early and late effector functions in response to FcεRI-mediated stimulation were not impaired in the absence of MAZR. Taken together, out data indicate that MAZR acts as a transcriptional repressor in mast cells, although MAZR does not play a major role in the transcriptional regulation of mast cell effector functions in response to FcεRI stimulation.

## Materials and Methods

### Ethics statement

All animal experiments were evaluated by the ethics committees of the Medical University of Vienna and approved by the Federal Ministry for Science and Research, Vienna, Austria (GZ:BMWF-66.009/0057-II/10b/2010 and GZ:BMWF-66.009/58-II/10b/2010). Animal husbandry and experimentation was performed under the national laws (Federal Ministry for Science and Research, Vienna, Austria) and ethics committees of the Medical University of Vienna and the University of Veterinary Medicine Vienna and according to the guidelines of FELASA which match that of ARRIVE.

### Mice

*Vav-iCre* ^*+*^ mice were kindly provided by D. Kioussis [18] and *Mazr* ^*F/F*^ *Vav-iCre* mice were backcrossed onto a C57BL/6 background (N>9). *Mcpt5-Cre* mice were previously described [19]. Mice analyzed were 8-12 weeks old unless otherwise stated. All mice were maintained in the preclinical research facility of the Medical University of Vienna. 

### Generation of bone marrow-derived mast cells (BMMC)

BM cells of the various genotypes were isolated from 8-12 weeks old mice. Femurs were dissected and BM cells were isolated by flushing the bones with mast cell medium: RPMI (Sigma) containing 10% heat-inactivated FBS (PAA), 5 µM β-mercaptoethanol (Gibco), penicillin/streptomycin (Gibco), 2 mM Glutamin (Gibco). Cell numbers were determined using CASY Counter (Schärfe-Systems, Germany). BM cells in the size range between 5-15 µM were cultured at a concentration of 0.5x10^6^/ml in medium supplemented with 5 ng/ml of recombinant murine IL-3 (Peprotech, UK). Once per week, in the course of 4-5 weeks, cells were spun down, counted and re-seeded at the density of 0.5x10^6^/ml. The purity of the BMMC population was monitored by FACS and cells were only used for assays if the c-kit^+^FcεRI^+^ mature BMMC constituted >95% of the total cultured cells.

### Genotyping of mice and BMMC

The genotype of mice used for BMMC isolation and *in vivo* experiments was determined by PCR of tail DNA. Bone marrow DNA and 5 week-cultured BMMC DNA was also extracted for confirmation of MAZR deletion by PCR. A detailed protocol can be obtained on request. The primer sequences used for genotyping are shown in Table S1.

### Toluidine blue staining of BMMC

A total of 0.3x10^5^ BMMCs was spun onto glass slides using a cytospin centrifuge (Shandon II) at 700 rpm for 3 min. Toluidine blue (Sigma) was dissolved in RNA-free water (PAA) at 10mg/ml. Slides were stained for 1 min in solution, rinsed in water and, once dry, embedded with Entellan mounting medium (Merck) and photographed using Nikon fluorescence microscope with CCD camera. 

### Flow cytometric analysis

The following antibodies were used for the flow cytometric analysis: CD11b (M1/70), CD11c (HL3), CD117 (c-kit; 2B8), FcεRI (MAR-1), Sca-1 (D7), CD34 (RAM34), CD49D (Integrin α4; R1-2) were obtained from BD Biosciences. IgE (23G-3) and CD123 (IL3Rα; 5B11) were obtained from eBiosciences. Measurements were collected with a flow cytometer (FACSCalibur or LSRII; BD Biosciences) and were analyzed with FlowJo software (TreeStar, Inc.). Cell sorting was performed on FACSAria (BD Biosciences). 

### Histological analysis

Whole left and right ears of sacrificed *Mazr* ^*F/F*^ and *Mazr* ^*F/F*^ *Vav-iCre* mice were excised and fixed for 30 min at RT in 4% PFA in PBS, followed by overnight incubation at 4°C. Ears were then dehydrated, embedded in paraffin, and cut onto 75 μm capillary gap microscope slides (Dako). Slides were then stained in 0.5% Toluidine Blue for 3 min, washed with running tap water and mounted with Entellan mounting medium. Mast cells were counted in 10 slides per mouse with steps of 10 µm from the previous section, and were photographed using Nikon fluorescence microscope with CCD camera at ×20 magnification. Printouts were coded before quantification to ensure unbiased counting of mast cells. 

### Degranulation of BMMC

Mature BMMC (>95% c-kit^+^FcεRI^+^) were primed (sensitized) in the following way: cells were spun down, counted and re-suspended at the concentration of 1x10^6^/ml in full mast cell medium containing 1 µg/ml of anti-TNP IgE mAb (#557079, BD Pharmingen) overnight. Next day 100 µl of cells (2.5x10^6^/ml) in Tyrodes buffer (10 mM HEPES pH 7.4, 130 mM NaCl, 5 mM KCl, 1.4 mM CaCl_2_, 1 mM MgCl_2_, 5.6 mM glucose, 0.1% BSA, sterile filtered) were activated with addition of 50 µl of Tyrodes buffer containing either 900 ng/ml of TNP(13)-BSA (final conc. 300 ng/ml) (Biosearch Technologies, Inc.), or with 105 ng/ml of PMA (final conc. 35 ng/ml) (Sigma-Aldrich) and 4500 ng/ml of Ionomycin (final conc. 1500 ng/ml) (Sigma-Aldrich) mix for 10 min at 37°C on 96 well plate followed by 5 min incubation on ice. Plates were then spun down for 4 min at 1400 rpm and supernatants were aliquoted in 50 μl duplicates on a fresh 96 well plate and 100 μl of 2 mM 4-Nitrophenyl N-acetyl-β-D-glucosaminide (Sigma) in 0.2 M citrate, pH 4.5 was added and color reaction was developed for 1 hour at 37°C. The remaining 50 µl of supernatant were carefully removed and cell pellet was lysed in 150 μl of 1% Triton X-100 in Tyrodes buffer for 15 min at RT. The lysates were aliquoted in 50 μl duplicates on a fresh 96 well plate and color reaction was developed as described for supernatants. The reaction was stopped by adding 150 μl of 1 M Tris-Cl pH 9, and the absorbance at 405 nm was measured in *Mithras* microplate reader (Berthold Technologies). OD values were normalized by subtracting the absorbance value of non-treated cell supernatants and the percent of β-hexosaminidase release was calculated using the following formula: release (%) = absorbance of SN / (absorbance of SN + absorbance of cell lysate) × 100.

### Cytokine measurements

24 well plates were coated overnight at 4°C with 0.5 ml of various concentrations of TNP-BSA in PBS. As it was not always possible to obtain the same conjugation of TNP to BSA from the provider, dose response curve was measured and TNP-BSA concentration was adjusted accordingly as the peak of the dose response. BMMC (2x10^6^ in 500 µl medium, 24 well plate) were sensitized overnight with anti-TNP IgE mAb as described above. TNP-BSA-coated plates were washed 3x in PBS, then blocked for 2 hours in 3% detoxified BSA at RT followed by 3 more washes in PBS. Cells were activated on the plates for 2 to 72 hours. Plates were spun down for 4 min at 1400 rpm and supernatants were collected. TNFα, IL-6, IL-4, IL-5 and GM-CSF cytokine levels in the supernatants were assessed by ELISA (BD Biosciences). Measurements were performed according to the manufacturer’s protocols. 

### Calcium flux in BMMC

Anti-TNP IgE overnight-primed cells (10^6^/ml) were labeled for 30 min at 37°C with 1 µM Indo-1 (Molecular Probes) and the Ca^2+^-dependent fluorescence of Indo-1 was assessed by flow cytometry. A total of 0.25x10^6^ cells were stimulated with 500 ng/ml TNP in 0.5 ml. Data were analyzed using FlowJo (TreeStar) software.

### Leukotriene B4 production

BMMC were sensitized with anti-TNP IgE mAb overnight. Next day 100 µl of cells in Tyrodes buffer (50x10^6^/ml) were activated with 10 µl of 1000 ng/ml TNP-BSA for 1 hour at 37°C. The LTB_4_ concentration was measured using EIA Kit (Cayman Chemical) according to manufacturer’s instructions. 

### Passive cutaneous anaphylaxis

Mice were anesthetized by intraperitoneal injection of 200-250 μl of Rompun (10 mg/kg)/Ketamine (100 mg/kg) mix. Left ears of mice were injected with 50 μl PBS, while right ears were injected with 0.5 μg of mIgE anti-TNP in 50 μl PBS. Next day mice were challenged intravenously with 250 μg of TNP-BSA in 150 μl PBS containing 1% Evans Blue dye (Sigma). After 4 hours mice were sacrificed, ears were excised and incubated in 500 μl of formamide for 48 hours at 55°C. Quantitative analysis of cutaneous anaphylaxis was conducted by measuring the absorbance of Evans Blue at 590 nm.

### Passive systemic anaphylaxis

Mice were intravenously injected with 2 μg of mIgE anti-TNP in 150 μl PBS. Next day mice were intravenously injected with either 500 μg of TNP-BSA in 150 µl of PBS or 150 μl of PBS only. After 3 minutes mice were sacrificed and blood was collected by cardiac puncture. Blood was then left to clot for 1 hour on ice, spun down and serum was collected. Histamine levels in serum were assessed by histamine ELISA (IBL International) following the manufacturer’s instructions.

### RNA extraction and quantitative real time PCR (qRTPCR) analysis

BMMC (at least 0.5x10^5^ in FACS buffer after FACS sorting, or 2x10^6^ in 2 ml of medium after activation) were lysed in TRIZOL reagent (Invitrogen) and total RNA was extracted according to the manufacturer’s instructions. RNA was reverse transcribed into cDNA using SuperScript II reverse transcriptase (Invitrogen) and expression levels of various genes was assessed via SYBR Green dye (Bio-Rad) incorporation in qRTPCR. *Hprt* or in some cases *Mazr* were used as input controls. The primer sequences used for qRTPCR are shown in Table S2.

### Retroviral-mediated expression of MAZR in BMMC

To generate retroviral supernatants, 3.5x10^6^ Phoenix-E cells were seeded on 10 cm culture dishes overnight in complete mast cell medium with addition of 2.5 U/ml of SCF, then were transiently transfected by CaCl_2_ and HEPES-buffered saline-mediated coprecipitation with 7 μg of the gag-pol-env-coding plasmid and 14 μg of the MIG-R-based retroviral constructs containing *Mazr* followed by an *IRES-EGFP* cassette that allows tracking of transduced cells with addition of 1 μl/ml of 50 mM chloroquine. Post-transfection cells were incubated at 37°C while the medium was changed 6, 30, and 48 hours post-transfection. Retrovirus-containing supernatant was collected gently by a syringe at 48 and 72 hours post-transfection, filtered through a 45 μm filter, and mixed with polybrene (4 μg/ml final concentration). Mature *Mazr* ^F/F^ and *Mazr* ^*F/F*^ *Vav-iCre* BMMC were then spin infected twice with 24 hours interval (2 hours, 1400 rpm at 32°C). As a control, *Mazr* ^F/F^ and *Mazr* ^*F/F*^ *Vav-iCre* BMMC were infected with an "empty" MIG-R constructs. Two days after infections, EGFP^+^ MIG-R- or MAZR-transduced BMMCs were FACS sorted and the expression of *Ccl5*, *Cccr5*, *Cxcl10*, *Il18*, *Arhgef18* and *Mazr* was determined by qRTPCR analysis. The retroviral transduction efficiency was between 0.2 - 3%.

### Mast cell starvation experiment

BMMC were generated as described above. On day 35 cells were counted by CASY counter and 25x10^6^ cells were split into fresh mast cell medium (0.5x10^6^/ml) that lacks IL-3, followed by a flow cytometric analysis of 1 ml of cells for c-kit, FcεRI and propidium iodide to assess the number of surviving mast cells in culture. The flow cytometric analysis was repeated for 5 consecutive days. The percentage of surviving mast cells was calculated as the relative number of c-kit^+^FcεRI^+^PI^-^ population. 

### Microarray analysis

RNA was isolated from IgE-primed mast cells (20x10^6^) using 1 ml Triazol. Gene expression studies were performed with Agilent Whole Genome Microarrays (Agilent’s SurePrint G3 Mouse GE 8x60K Microarray, Agilent Microarray Design ID 028005). The data analysis was performed using GeneSpring software 11.5 (Agilent Technologies). The cutoffs for differential expression were set as absolute fold-change >2 and a corrected P-value of < 0.1. Microarray data are available in the ArrayExpress database (www.ebi.ac.uk/arrayexpress) under accession number E-MTAB-1816.

### Statistical analysis

Unless otherwise stated the data are presented as mean ± SEM. GraphPad Prism software was used for data analysis and plotting. The P-values were calculated with two-tailed unpaired sample Student’s T-test where significance levels were set as following: *, P < 0.05; **, P < 0.01; ***, P < 0.001; n.s., not significant.

## Results

### Generation of conditional MAZR-deficient mice

To overcome limitations in the analysis of *Mazr* ^*-/-*^ mice due to embryonic lethality, we generated a conditional "floxed" *Mazr* allele using the same targeting strategy as previously described [8] (Figures S1A and S1B). *Mazr* ^F/F^ mice did not show any developmental or growth defects and *Mazr* ^F/F^ mice did not show alterations in the CD4/CD8 ratio as described for *Mazr* ^*-/-*^ mice (data not shown; [8]), indicating that the inserted loxP sites did not interfere with MAZR expression. To study the role of MAZR in mast cells, we crossed *Mazr* ^F/F^ mice with the *Vav-iCre* deleter strain, which is active in all hematopoietic cells [18]. *Mazr* ^*F/F*^ *Vav-iCre* mice did not show any developmental or growth defects in comparison to *Mazr* ^*F/F*^ mice (Figures S2A and S2B). Total BM cells as well as BM-derived mast cells from *Mazr* ^*F/F*^ *Vav-iCre* mice showed efficient deletion of the floxed *Mazr* alleles (Figure S3A). Thus, as suggested by another study [10], the loss of MAZR in hematopoietic cells is not the cause of the observed embryonic lethality in *Mazr* ^*-/-*^ mice. 

### MAZR is required for the efficient generation of BMMCs *in vitro*

**Figure 1:**
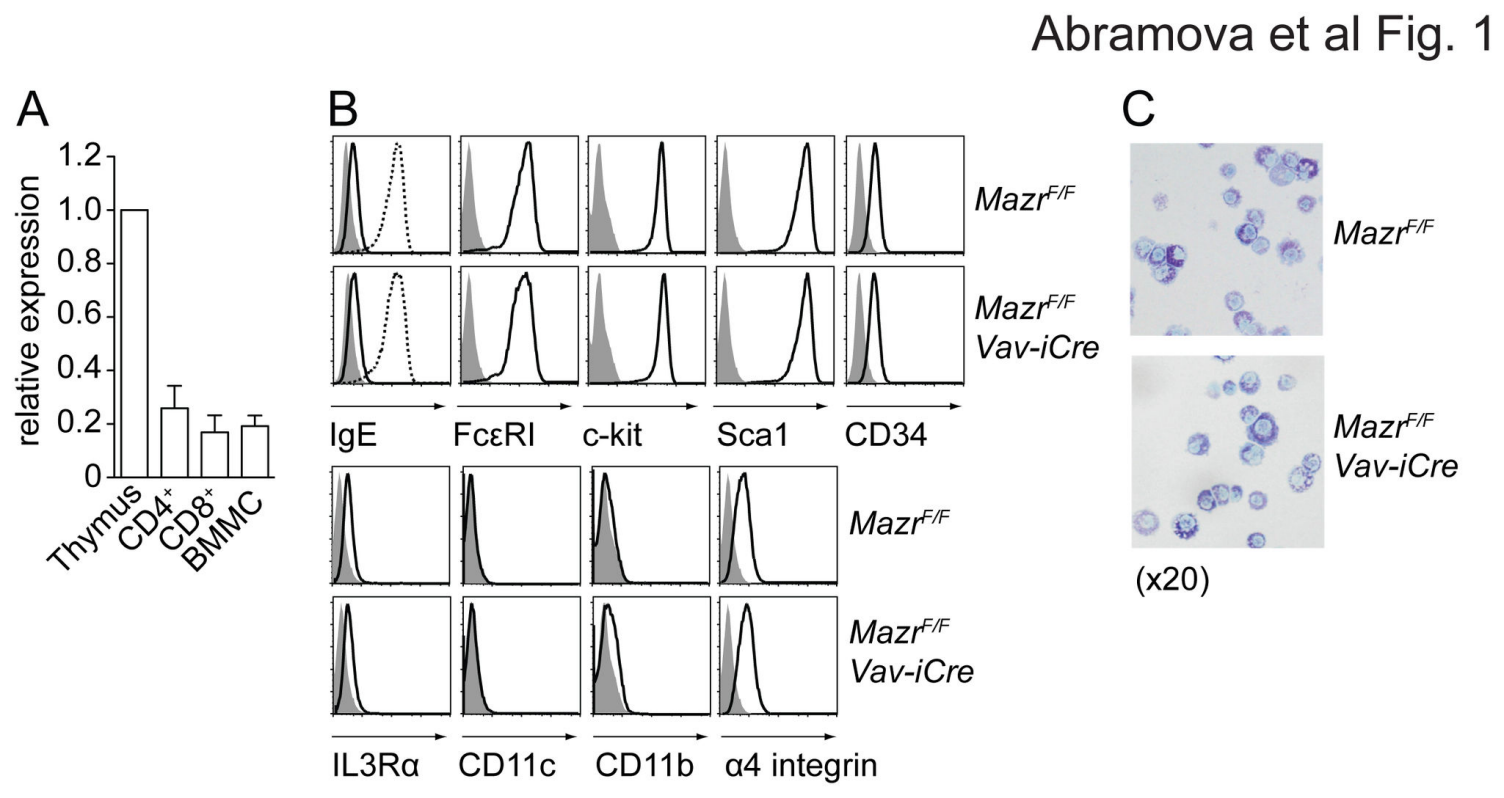
MAZR is not essential for the differentiation of BM-derived mast cells. (A) Diagram shows qRTPCR analysis of *Mazr* expression in thymus, CD4^+^ and CD8^+^ T cells and in IgE-primed BM-derived mast cells (BMMC). Expression levels are normalized to *Hprt* expression and levels in thymocytes were set as 1 (100%). Columns represent a summary of three independent samples. Mean ± SEM is shown. (B) Histograms depict expression of cell surface markers on *Mazr* ^F/F^ and *Mazr* ^*F/F*^ *Vav-iCre* BMMC (after 5 weeks of culture). Filled gray areas are isotype control stainings. Data are representative of three independent experiments. (C) Flow cytometric analysis showing up-regulation of FcεRI levels in *Mazr* ^F/F^ and *Mazr* ^*F/F*^ *Vav-iCre* BMMC. Filled gray areas are isotype control stainings. The solid black line shows the levels of cell-surface bound IgE after 15 min incubation. The dotted line shows the levels of cell-surface bound IgE after overnight priming. Data are representative of three independent experiments. (D) Toluidine blue staining of 5 week-cultured *Mazr* ^F/F^ and *Mazr* ^*F/F*^ *Vav-iCre* BMMC prepared by a cytospin. Magnification 20x.

**Figure 2:**
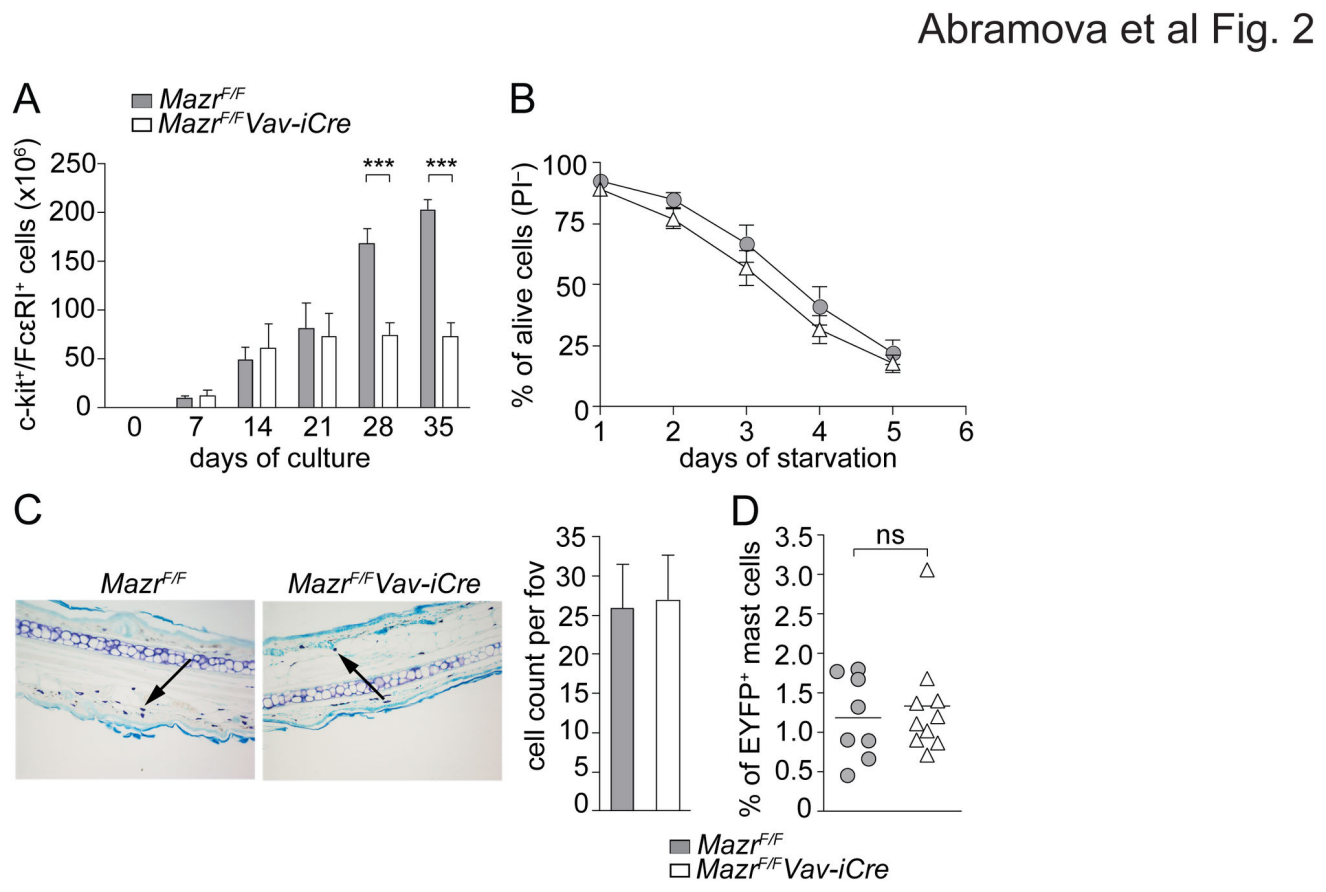
Reduced mast cell numbers *in vitro* but normal mast cell homeostasis *in vivo*. (A) Diagram showing the cumulative numbers of c-kit^+^FcεRI^+^ *Mazr* ^F/F^ and *Mazr* ^*F/F*^ *Vav-iCre* BMMC over the course of 5 weeks of culture. Cells were counted by CASY counter, then percentages of c-kit^+^FcεRI^+^ BMMC among PI-negative cells (= alive) was determined by flow cytometry. The summary of three independent experiments with a total of 6 independent cell batches is shown. Mean ± SEM is shown. (B) Number of PI-negative (= alive) *Mazr* ^F/F^ and *Mazr* ^*F/F*^ *Vav-iCre* BMMC during 5 days of IL-3 starvation. The summary of 3 experiments is shown. Mean ± SEM is shown. (C) Toluidine blue staining of paraffin-embedded 5 µm ear sections of *Mazr* ^F/F^ and *Mazr* ^*F/F*^ *Vav-iCre* mice showing mast cells in pink/purple color (examples indicated by arrowheads). Diagram at the right indicates mean ± SEM of mast cell number per field of view (fov) calculated over 10 individual sections per ear (n=4). Magnification 20x. (D) Percentage of EYFP^+^c-kit^+^FcεRI^+^ mast cells from peritoneal lavage of wild-type (Mazr^F/+^Rosa26^+/EYFP^Mcpt5Cre) and mast cell-specific MAZR-null (Mazr^F/F^Rosa26^+/EYFP^Mcpt5Cre) mice (n=8 and 9, respectively).

In order to study whether MAZR is essential for the generation of BMMCs, *Mazr* ^F/F^ and *Mazr* ^*F/F*^ *Vav-iCre* BM cells were cultured in the presence of IL-3 for 4-6 weeks as described previously [20]. Quantitative RTPCR analysis confirmed the expression of *Mazr* in IgE-primed BMMC (Figure 1A), although MAZR protein expression in BMMCs was not detected in comparison to thymocytes (data not shown), most likely due to the lower mRNA expression levels relative to thymocytes (Figure 1A). A comprehensive analysis of several cell surface markers expression revealed no phenotypic differences between wt and MAZR-null BMMCs (Figure 1B) and FcεRI levels were up-regulated to a similar extent upon overnight incubation with IgE (Figure 1C). Moreover, toluidine blue stainings revealed no detectable morphological differences between *Mazr* ^F/F^ and *Mazr* ^*F/F*^ *Vav-iCre* BMMCs (Figure 1D). In addition, *Mazr* alleles were efficiently deleted in BMMCs (Figures S3A and S3B), indicating that there is no mast cell population present in the culture that has escaped deletion of one of the *Mazr* alleles. We noted, however, that *Mazr* ^*F/F*^ *Vav-iCre* BM cultures yielded less BMMCs in comparison to *Mazr* ^*F/F*^ BM cultures. To analyze this in more detail, *Mazr* ^F/F^ and *Mazr* ^*F/F*^ *Vav-iCre* BM cultures were quantitatively monitored over a period of 5 weeks for the appearance of c-kit^+^FcεRI^+^ mast cells in the presence of IL-3. While *Mazr* ^F/F^ mast cell numbers steadily increased over a period of 5 weeks, *Mazr* ^*F/F*^ *Vav-iCre* BMMC numbers peaked at around 3 weeks and remained constant over the next 2 weeks (Figure 2A). Connective tissue-type BMMC generated in the presence of IL-3 and SCF showed a similar reduction in cell numbers in the absence of MAZR (data not shown). The reduced numbers of IL-3-generated BMMC, however, were not due to a general reduced cell survival or an increase in cell death, since the number of propidium iodide-negative (PI^-^) cells (i.e. alive cells) during regular cell culture was similar between *Mazr* ^F/F^ and *Mazr* ^*F/F*^ *Vav-iCre* BMMC (data not shown) and *Mazr* ^F/F^ and *Mazr* ^*F/F*^ *Vav-iCre* BMMC showed a similar survival kinetic after 5 days of IL-3 starvation (Figure 2B). 

 To test whether reduced numbers of mast cells were also observed *in vivo*, mast cell numbers in the skin of the ear and in the peritoneum were determined. In contrast to *in vitro*-derived mast cells, the *in vivo* numbers of mast cells in the ear skin were similar in *Mazr* ^F/F^ and *Mazr* ^*F/F*^ *Vav-iCre* mice (Figure 2C). For the determination of peritoneal mast cell numbers, *Mazr* ^*F/F*^ mice were crossed with the *Mcpt5Cre* deleter strain, a BAC transgenic line in which Cre expression is driven by the mast cell protease 5 promoter [19]. This line was shown to efficiently delete, among other mast cell subsets, in peritoneal mast cells [19]. To reveal mast cells that have active Cre, the mice were also crossed onto a *Rosa26-EYFP* reporter allele (*Rosa26* ^*+/EFYP*^) that expresses EYFP upon Cre-mediated deletion (due to deletion of a transcriptional stop cassette in front of the EYFP coding sequence) [21]. Wild-type (*Mazr* ^*F/+*^ *Rosa26* ^*+/EYFP*^ *Mcpt5Cre*) and mast cell-specific MAZR-null (*Mazr* ^*F/F*^ *Rosa26* ^*+/EYFP*^ *Mcpt5Cre*) displayed similar percentages of EYFP^+^c-kit^+^FcεRI^+^ peritoneal mast cells (Figure 2D) and the fraction of c-kit^+^FcεRI^+^ cells within the EYFP^+^ cell population was similar (Figures S4A and S4B). This indicates normal numbers of peritoneal mast cells under homeostatic conditions. Together, these data suggest that MAZR is required for the efficient generation of mast cells *in vitro*, although mast cell numbers *in vivo* appear to be normal in the absence of MAZR.

### Gene expression data reveal that MAZR acts as a transcriptional repressor in mast cells

**Figure 3:**
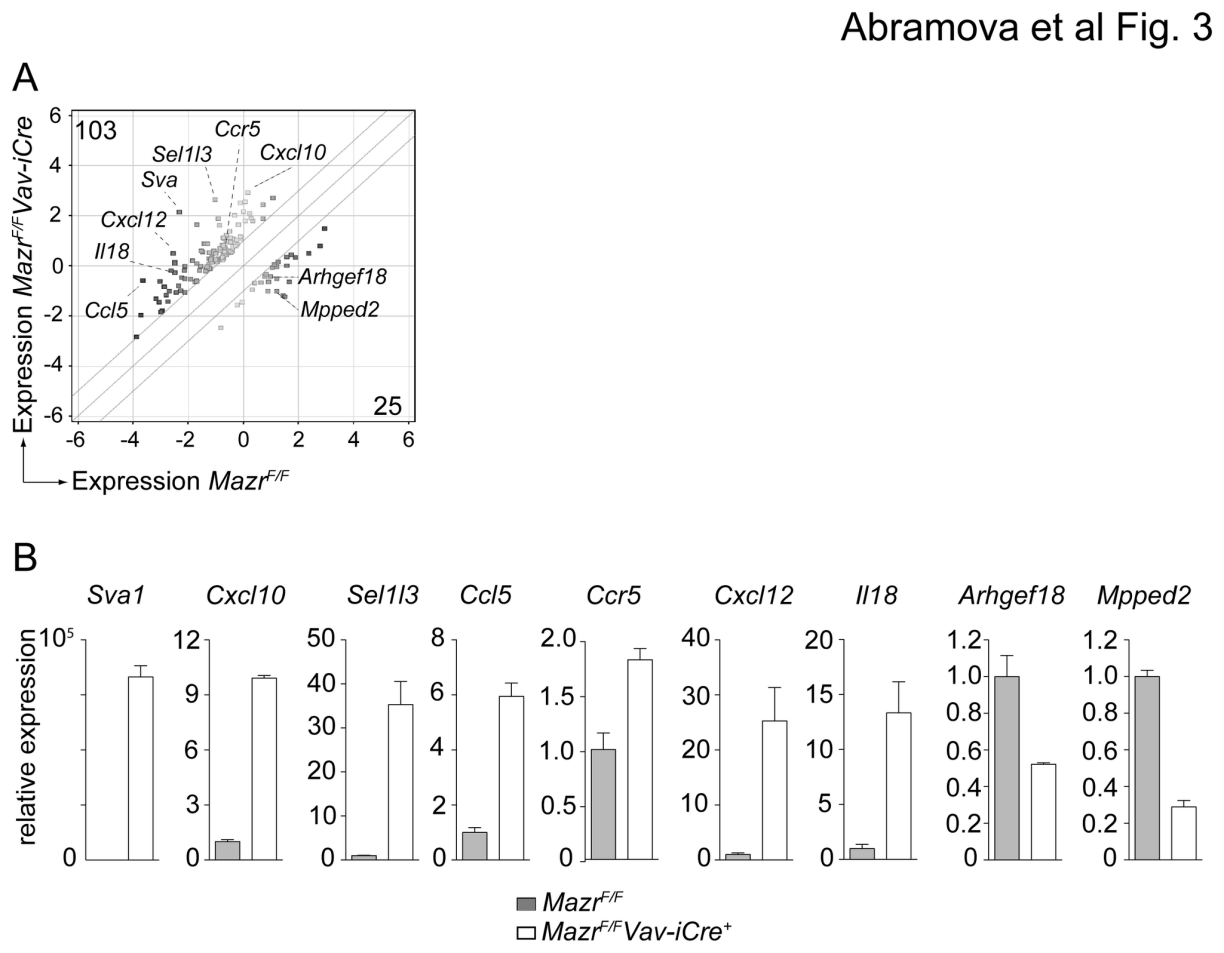
Gene expression analysis of non-activated MAZR-deficient BMMCs. (A) Gene expression profiles from IgE-primed (but non-activated) *Mazr* ^F/F^ and *Mazr* ^*F/F*^ *Vav-iCre* BMMC were determined using Agilent arrays. Data were analyzed using GeneSpring software as described in materials and methods. The scatter plot indicates the 128 genes that are dysregulated in the absence of MAZR (log_2_ expression levels; ≥2 fold-difference, P≤0.1). Numbers at the upper-left or lower-right corners show the number of genes specifically expressed in *Mazr* ^*F/F*^ *Vav-iCre* (Y-axis) and *Mazr* ^F/F^ (X-axis) BMMCs. The highlighted genes are selected from the top-ten hits with the largest-fold difference between *Mazr* ^F/F^ and *Mazr* ^*F/F*^ *Vav-iCre* BMMC. (B) qRTPCR analysis of genes selected from the microarray experiments. Graphs represent relative expression levels of genes up- and down-regulated in the absence of MAZR (normalized to *Hprt*). Expression levels in *Mazr* ^F/F^ samples were set to 1. Mean ± SEM is shown. Data are means of the results from duplicated qRTPCRs of one batch of mast cells. The IgE-primed *Mazr* ^F/F^ and *Mazr* ^*F/F*^ *Vav-iCre* BMMC samples were from a different batch compared to the ones used for probing with Agilent arrays.

To identify pathways that might be affected by loss of MAZR and that might explain the reduced cells numbers observed *in vitro*, the gene expression profiles of *Mazr* ^F/F^ and *Mazr* ^*F/F*^ *Vav-iCre* of IgE-primed BMMC were determined using Agilent arrays. This revealed that 103 genes were up- and 25 genes were down-regulated in the absence of MAZR (fold change ≥ 2; P < 0.1) (Figure 3A, Table S3). The genes identified by Agilent arrays were classified according to the biological process and their molecular function (using DAVID functional annotation bioinformatics microarray analysis; http://david.abcc.ncifcrf.gov; [22,23]) (Table S4). The biological processes with the most up-regulated genes in the absence of MAZR were “ion transport and cellular homeostasis” and “inflammatory/immune responses” (Table S4). For down-regulated genes, the largest class was “sensory perception/neurological systems process/G-protein coupled receptor signaling”. Among the genes that were up-regulated in the absence of MAZR were several chemokines (*Ccl5*, *Cxcl10*, *Cxcl12*), chemokine receptor *Ccr5* and cytokine *Il18*. The differential expression of some of the identified genes was also confirmed by qRTPCR analysis of IgE-primed *Mazr* ^F/F^ and *Mazr* ^*F/F*^ *Vav-iCre* BMMC (Figure 3B). These results suggest an immunoregulatory role of MAZR in mast cells. Since a larger number of genes was up- than down-regulated in the absence of MAZR, our data also indicate that MAZR preferentially acts as a transcriptional repressor in mast cells. 

**Figure 4:**
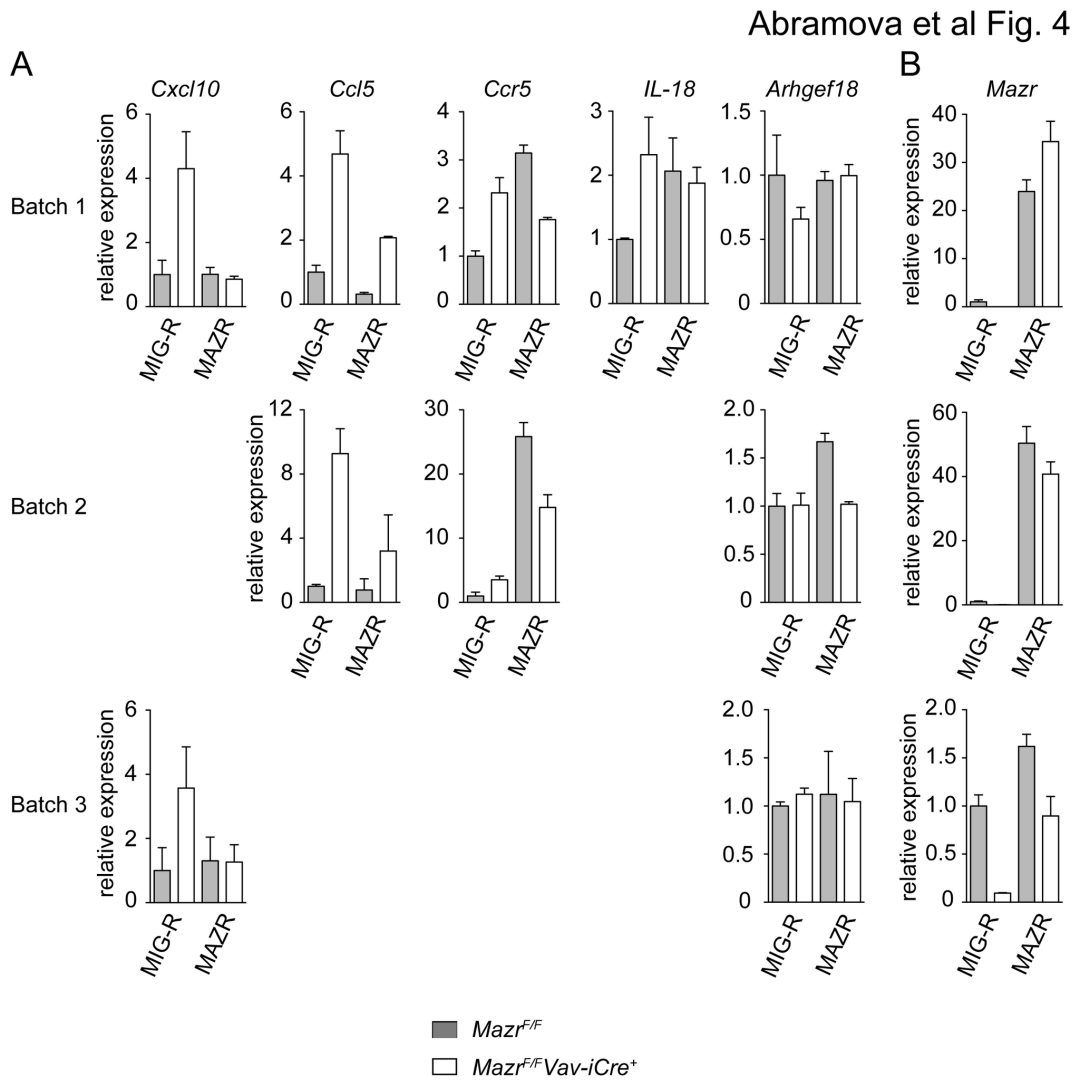
Enforced expression of MAZR in MAZR-null BMMCs partially rescues altered gene expression patterns. (A) *Mazr* ^F/F^ and *Mazr* ^*F/F*^ *Vav-iCre* BMMC were cultured for 5 weeks. Subsequently, cells were retrovirally transduced with MAZR or with the parental MIG-R vector (containing only IRES-EGFP). Two days later, EGFP^+^ cells were sorted, RNA isolated and the expression levels of the indicated genes were determined by qRTPCR. Expression levels in each sample were normalized to *Hprt* expression. Expression in MIG-R-transduced *Mazr* ^F/F^ mast cells was set as 1. The results of three independent transduction experiments (batch 1, 2 and 3) are shown. (B) Endogenous (for MIG-R transduced) and exogenous (for MAZR-transduced) *Mazr* expression levels of transduced mast cells (batch 1, 2 and 3) was determined by qRTPCR. Expression in MIG-R-transduced *Mazr* ^F/F^ mast cells was set as 1. (A, B) qRTPCR for each batch was performed in duplicates. Mean ± SEM is shown.

Next, we wanted to test whether the altered gene expression patterns in the absence of MAZR can be rescued upon re-expression of MAZR. Therefore, *Mazr* ^F/F^ and *Mazr* ^*F/F*^ *Vav-iCre* BMMC were retrovirally transduced in two rounds of spin infection with MIG-R-based retroviral constructs containing *Mazr* followed by an *IRES-EGFP* cassette that allows tracking of transduced cells. As a control, *Mazr* ^F/F^ and *Mazr* ^*F/F*^ *Vav-iCre* BMMC were infected with an "empty" MIG-R construct. Two days after infections, EGFP^+^ MIG-R- or MAZR-transduced BMMCs were sorted and the expression of a few selected genes obtained from the array data (*Ccl5*, *Ccr5*, *Cxcl10*, *Il18* and *Arhgef18*) was determined by qRTPCR analysis. This revealed that the altered expression of *Ccl5* and *Cxcl10* in *Mazr* ^*F/F*^ *Vav-iCre* BMMC was rescued to wild-type levels upon enforced expression of MAZR (Figure 4A). Exogenous *Mazr* was overexpressed 30-40 fold over wild-type levels in batch 1 and 2, while *Mazr* expression levels were similar to wild-type levels in batch 3 (Figure 4B). Of note, *Arhgef18*, which was approx. 2.6-fold down-regulated in IgE-primed MAZR-null BMMCs (Table S3) was not down-regulated in retrovirally transduced non-primed BMMCs (Figure 4A).

### Early and late effector functions in MAZR-deficient mast cells

**Figure 5:**
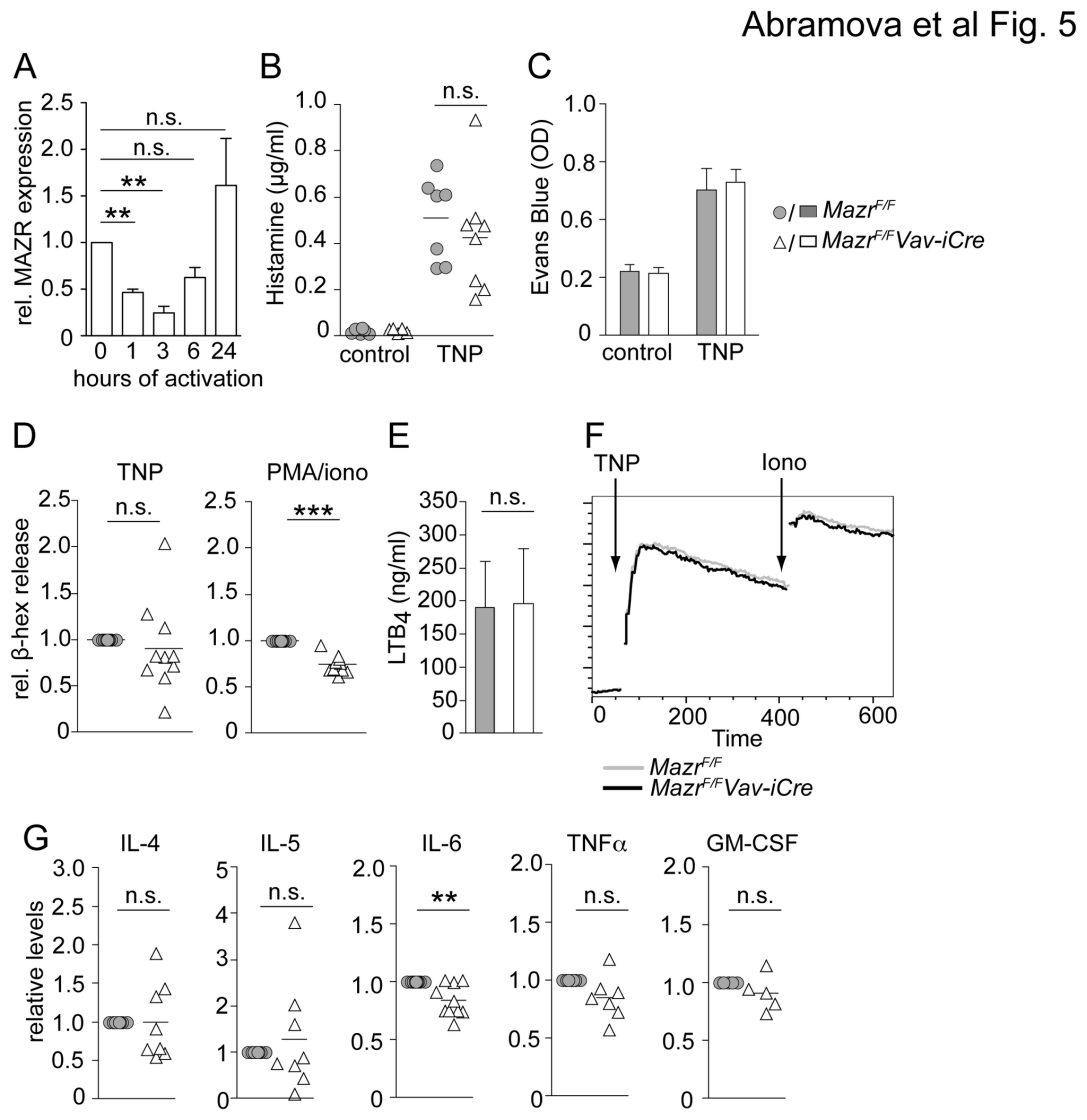
Minor defects in early and late mast cell effector functions in the absence of MAZR. (A) Diagram shows qRTPCR analysis of *Mazr* expression in resting anti-TNP IgE-primed BMMCs and in BMMCs activated for the indicated time points with TNP. Expression levels are normalized to *Hprt* expression and levels in IgE-primed non-activated mast cells were set as 1 (100%). Data show summary of three samples analyzed. Mean ± SEM is shown. (B) Plasma histamine levels in a systemic anaphylaxis model are shown. *Mazr* ^F/F^ and *Mazr* ^*F/F*^ *Vav-iCre* mice were primed (i.v.) with anti-TNP IgE, challenged 24 hours later by i.v. injection of TNP or PBS. Serum was collected 2 minutes post-injection and histamine levels were determined by ELISA, n=7. (C) Absorbance (OD) of Evans Blue dye extravasated in a passive cutaneous anaphylaxis model from the ears of *Mazr* ^F/F^ and *Mazr* ^*F/F*^ *Vav-iCre* mice is shown. Mice were injected with PBS and anti-TNP IgE into left and right ear, respectively, and 24 hours later mice were challenged by i.v. injection with TNP/Evans Blue dye. Extravasation of Evans Blue dye in the ear was measured 4 hours later. Diagram shows summary of 9 mice. Mean ± SEM is shown. (D) Anti-TNP-IgE-primed *Mazr* ^F/F^ and *Mazr* ^*F/F*^ *Vav-iCre* BMMCs were activated for 10 min with TNP or PMA/ionomycin. β-Hexosaminidase release levels of *Mazr* ^*F/F*^ BMMCs were set to 1 (n=10). (E) Anti-TNP-IgE-primed *Mazr* ^F/F^ and *Mazr* ^*F/F*^ *Vav-iCre* BMMCs were activated for 60 min with TNP. LTB_4_ levels were determined by ELISA. Mean ± SEM is shown. (n=4). (F) Flow cytometric analysis of calcium flux in anti-TNP-IgE-primed *Mazr* ^F/F^ and *Mazr* ^*F/F*^ *Vav-iCre* BMMCs that have been activated with TNP. Data are representative of 3 independent experiments. (G) Cytokine production of anti-TNP-IgE-primed *Mazr* ^F/F^ and *Mazr* ^*F/F*^ *Vav-iCre* BMMCs that were activated by plate-bound TNP for 24 hours (at least 5 independent mast cell batches were analyzed). Cytokine production of *Mazr* ^*F/F*^ BMMCs was set to 1.

In a next step we wanted to investigate whether the transcriptional changes caused by loss of MAZR under steady state conditions in IgE-primed BMMCs led to alterations in early and late mast cells effector functions in response to FcεRI-mediated activation. We initially tested by qRTPCR whether MAZR expression is modulated upon FcεRI-triggering. Upon activation, *Mazr* was transiently down-modulated during the first 3 hours after activation, however, after 24 hours, *Mazr* expression went back to levels that were even slightly higher than the ones observed in non-stimulated mast cells, although the increase in *Mazr* levels over non-activated cells was not statistically significant (Figure 5A). This revealed a dynamic expression pattern of MAZR during mast cell activation.

 To test early effector functions of MAZR-deficient mast cells *in vivo*, we employed systemic and cutaneous anaphylactic reaction models. For systemic anaphylaxis, *Mazr* ^F/F^ and *Mazr* ^*F/F*^ *Vav-iCre* mice were primed with murine IgE specific for TNP by an i.v. injection, 24 hours later mice were challenged by an i.v. injection of TNP-BSA and histamine levels in the serum were determined. Histamine ELISA showed that *Mazr* ^F/F^ and *Mazr* ^*F/F*^ *Vav-iCre* mice have released similar levels of histamine (Figure 5B), suggesting normal histamine release response of mast cells *in vivo*. In cutaneous anaphylaxis model, *Mazr* ^F/F^ and *Mazr* ^*F/F*^ *Vav-iCre* mice displayed similar levels of Evans Blue dye extravasation in ears, indicating similar levels of cutaneous anaphylactic response (Figure 5C). To further study mast cell *in vitro*, *Mazr* ^*F/F*^ and *Mazr* ^*F/F*^ *Vav-iCre* BMMC were loaded overnight with IgE specific for TNP and subsequently primed cells were stimulated with TNP or PMA/ionomycin. This revealed that *Mazr* ^*F/F*^ and *Mazr* ^*F/F*^ *Vav-iCre* mast cells released similar amount of β-hexosaminidase (Figure 5D), although there was a tendency that degranulation was slightly reduced in the absence of MAZR, in particular upon activation with PMA/ionomycin (Figure 5D). However, leukotriene LTB_4_ secretion and Ca^2+^ mobilization was normal in MAZR-null BMMC (Figure 5E and F), indicating that MAZR is not essential for early effector mast cell functions *in vitro* and *in vivo*. To determine whether MAZR is important for late effector functions, IgE-primed *Mazr* ^F/F^ and *Mazr* ^*F/F*^ *Vav-iCre* BMMC were activated with TNP for 24 hours and cytokine secretion was determined by ELISA. This revealed that IL-6 levels were slightly reduced in MAZR-null BMMCs and there was a tendency (with a P-value of 0.0764) that MAZR-null BMMC also produced less TNFα as compared to wt BMMC (Figure 5G). However, IL-4, IL-5 and GM-CSF levels were similar between *Mazr* ^F/F^ and *Mazr* ^*F/F*^ *Vav-iCre* BMMC (Figure 5G). Together, these results indicate that MAZR is not essential for early and late effector functions upon FcεRI-mediated activation of BMMC.

## Discussion

In this study we report the generation of a conditional *Mazr* allele to study the role of MAZR in mast cells. We demonstrate that in the absence of MAZR the expression of 128 genes is altered and that MAZR preferentially acts as a transcriptional repressor for the majority of these genes. In addition, despite modulated *Mazr* expression upon FcεRI-mediated stimulation, MAZR plays only a minor role in the transcriptional networks in mast cells that regulate early and late effector functions in response to FcεRI stimulation.

 The first important result of our study was the successful generation of a conditional *Mazr* allele. In addition to changes in CD4/CD8 cell fate choice of DP thymocytes, germ-line deletion of MAZR leads, dependent on the genetic background, to severely reduced numbers of or even no alive born pups [8] [10,11], thus conditional targeting strategies are required to study MAZR function. Moreover, the few surviving MAZR-null mice show severe growth retardation and reach only approximately 60% of the size of wild-type control mice [8,10,11]. Therefore, the conditional *Mazr* allele reported in this study represents a great and novel tool to overcome the phenotype of the germ-line MAZR knockout and to reveal the physiological function of MAZR in various cell lineages and tissues. Since *Mazr* ^*F/F*^ mice that have been crossed with the hematopoietic lineage-specific deleter strain *Vav-iCre* [18] were born at normal Mendelian ratio and did not display any obvious developmental and/or growth defects, our study also reveals that the observed embryonic lethality in germline MAZR knockout mice is not due to loss of MAZR in hematopoietic cell lineages.

 Although MAZR is expressed in mast cells, our study showed that mast cell development was not dependent on MAZR. There was no difference in the phenotypic appearance of BM-derived wild-type and MAZR-null mast cells with respect to morphology and the expression of several cell surface markers including c-kit and FcεRI. Moreover, mast cell numbers in the skin of the ear and in the peritoneum were normal in the absence of MAZR, although the number of *in vitro* generated BM-derived mast cell was reduced in the absence of MAZR. The difference between similar *in vivo* numbers and reduced *in vitro* numbers is likely to be explained by different growth requirements, e.g. it has been shown that IL-3 is not essential for the generation of mast cells under physiological conditions [24], while IL-3 strongly promotes mast cell growth *in vitro*. Nevertheless, our data indicate that MAZR is required for optimal generation of BMMC under *in vitro* growth/differentiation conditions. One possibility might be an altered frequency of mast cell progenitors in the absence of MAZR, which is revealed under *in vitro* culture conditions. It is also possible that other cell subsets influence the generation of BMMCs upon deletion of MAZR. MAZR is also implicated in cell cycle regulation and the induction of cellular senescence in murine embryonic fibroblasts [10,25], thus similar pathways might be affected in MAZR-null BMMCs. However, it is unlikely that the reduced yield in cell numbers was due to impaired survival of MAZR-deficient mast cells *in vitro*, since *Mazr* ^F/F^ and *Mazr* ^*F/F*^ *Vav-iCre* BMMC survived with a similar kinetic upon IL-3 withdrawal. Additional experiments are required to understand why mast cell numbers are reduced in the absence of MAZR. Of note, there are other examples of knockout mast cells that showed differences in BMMC numbers *in vitro* while similar numbers of mast cell numbers are observed *in vivo*, such as Btk-deficient [26] or Tec-deficient [20] mast cells, both of which display enhanced cell numbers in comparison to wild-type BMMC cultures. 

 Our study also revealed that MAZR functions as a transcriptional regulator for a limited number of genes in IgE-primed mast cells. The expression of 128 genes was altered in the absence of MAZR among which 103 genes were up-regulated and 25 were down-regulated. This clearly demonstrates that MAZR acts preferentially as a transcriptional repressor in mast cells. The transcriptional repression function of MAZR is in agreement with the studies performed in T cells, where it was shown that MAZR repressed *Cd8* genes in DN thymocytes [7], as well as *Thpok* gene expression in MHC class I-signaled DP thymocytes [8]. It is not known whether a similar number and types of genes are dysregulated in the absence of MAZR in other cell lineages, since no microarray data are published from other cell types that are MAZR-deficient. Based on DAVID bioinformatics tools (http://david.abcc.ncifcrf.gov; [22,23]) the genes dysregulated in the absence of MAZR could be grouped into several functional clusters with a broad range of biological functions (Table S4). The highest enrichment scores were observed for the GO categories “Locomotory behavior/chemotaxis”, “Inflammatory/Immune response” and “Positive regulation of immune responses” (Table S4). One large group of MAZR-regulated genes that falls into “Inflammatory/Immune responses” included cytokines, chemokines and chemokine receptors such as (*Ccl5, Cxcl12, Cxcl10, Il18, Clcf1, Tgfb2, Ltbr, Il1a, Ccr5*) suggesting that MAZR-deficient mast cells might have altered immunoregulatory functions. Of note, MAZR interacts with MITF and it has been proposed that both genes cooperate in the regulation of *Tpsb2* (encoding Tryptase beta 2) expression [12]. However, *Tpsb2* expression was not altered in the absence of MAZR. Moreover, we did not observe a dysregulation of the genes in MAZR-null BMMCs that were reported as differentially expressed in IL-3/SCF-generated connective tissue type BMMCs in the absence of MITF [15]. This suggests that loss of MAZR and MITF leads to different changes in gene expression patterns in mast cells, although we cannot exclude that MITF might share some target genes with MAZR in IL-3-generated mucosal type BMMCs. Moreover, other factors shown to interact with MITF and to modulate its transcriptional activity, such as PIAS3 [27] or Hint-1 [28] might function differentially in MAZR-null mast cells and thus might allow MITF to compensate for loss of MAZR [16].

 The dysregulation of gene expression in the absence of MAZR raises the question whether some of these genes are direct MAZR target genes. Our observation that enforced MAZR expression in MAZR-deficient mature BMMCs reverted the altered expression of some of the few genes tested within 48 hours post-transduction suggests that some of the dysregulated genes might be direct MAZR target genes. However, one gene tested (*Ccr5*) followed a completely different expression pattern upon enforced expression of MAZR, indicating that MAZR might affect gene expression in different ways. It is conceivable that the kinetic of transcriptional regulation by MAZR might be different for different target genes. Moreover, some of the genes might be indirectly regulated by MAZR (i.e. the gene is not a direct MAZR target gene). In preliminary ChIP assays binding of MAZR within a 1kb promoter proximal region could be detected only for *Il18* but not for the *Cxcl10*, *Cxcl12* and *Ccl5* gene loci (data not shown). However, MAZR might bind gene specific enhancers or other *cis*-regulatory elements as observed in T cells for the *Cd8ab1* gene complex [7] and *Thpo*k [8]. Thus, in future experiments genome-wide ChIP-seq experiments are required to determine MAZR binding sites in mast cells and to identify direct MAZR target genes. 

 The last finding of our study demonstrated that MAZR is not essential for FcεRI-mediated mast cell activation *in vitro* and *in vivo*, despite a modulated expression pattern of MAZR in response to FcεRI stimulation. There were no major differences in the early- and late effector functions of MAZR-null BMMCs, although IL-6 was reduced and there was a tendency that β-hexosaminidase release (in particular to PMA/ionomycin stimulation) and TNFα production were also slightly reduced in the absence of MAZR. Notably, the microarray data indicated that several factors implicated in the activation of small GTPases such as guanine nucleotide exchange factors (GEF) *Rasgrf1*, *Fgd4, Rapgef3* and *Arhgef18* (the first two were up-, while the latter two were down-regulated) and GTPase-activating protein (GAP) *Sgsm1* are dysregulated in the absence of MAZR. Since GTPases regulate a variety of cellular processes (dependent of the subfamily of GTPases) including endocytosis/exocytosis, signaling and migration, it is tempting to speculate that certain intracellular transport processes might be slightly altered upon loss of MAZR, leading to subtle changes in the secretion of the preformed mediator β-hexosaminidase. Even though the array analysis also suggested an altered immunoregulatory role of MAZR-deficient mast cells due to the dysregulated expression of certain chemokines and cytokines in non-stimulated mast cells, *in vivo* FcεRI-mediated mast cell functions in systemic and cutaneous anaphylactic reactions were not altered. Several reasons that are not mutually exclusive can be envisaged that might explain why MAZR-deficient mast cells display no *in vivo* alterations. Since MAZR is transiently down-regulated upon FcεRI-mediated activation, loss of MAZR activity might rather affect very late-phase effector functions *in vivo*. These functions might not be revealed within the 4 hours time-frame of the applied cutaneous anaphylaxis model, although MAZR-null BMMCs had approx. 50 genes dysregulated after a 4 hour FcεRI-mediated activation period in comparison to activated wt BMMCs (23 of these genes were already dysregulated in non-activated cells) (data not shown). Moreover, mast cell differentiation and activation in the absence of MAZR *in vivo* might lead to different gene expression patterns than the one observed in MAZR-null BMMCs, most likely due to the cytokine milieu and other local environmental factors that are known to influence mast cell activation and differentiation [1,5]. Finally, it is also possible that other transcription factors are up-regulated during (late) mast cell activation that can compensate for the loss of MAZR. In future experiments it will be important to employ alternative *in vivo* models (such as contact hypersensitivity models) for the assessment of mast cell function in the absence of MAZR.

Taken together, we have shown that the transcription factor MAZR preferentially acts as a transcriptional repressor in mast cells. However, MAZR plays only a minor role in the transcriptional networks in mast cells that regulate early and late responses to FcεRI stimulation. Future studies that investigate other mast cell activation pathways (e.g. TLR-mediated pathways) and functions are necessary to reveal potential roles of MAZR in mast cells and the consequences of the dysregulated gene expression in the absence of MAZR.

## Supporting Information

Figure S1 **Generation of a conditional *Mazr* allele.** (A) Schematic map of MAZR (641 aa) showing the N-terminal BTB domain and the 7 zinc fingers at the C-terminus.
(B) Targeting strategy for *Mazr* (as previously shown in [8]). Schematic map of the targeting construct (top panel), the endogenous *Mazr* gene locus (middle panel), the targeted *Mazr* gene locus after homologous recombination (*Mazr* ^*+/Fneo*^; upper bottom panel), and the targeted locus after the neomycin cassette deletion (*Mazr* ^*+/F*^; middle bottom panel) and iCre recombinase-mediated deletion of exon 1 (*Mazr* ^*+/Δ*^; lower bottom panel, respectively). All EcoRI (E), XhoI (X) and EcoRV (EV) restriction sites are shown. Open arrowheads (1, 2, and 3) indicate the location of the PCR primers used to detect deletion of exon 1. The horizontal thick black line (in the top and bottom panel) indicates the region of homology between the targeting construct and the endogenous locus. The thick bar (in the middle part) represents the 5' probe used for Southern-blotting. Horizontal bars with numbers (indicating the size in kb) show the expected genomic fragments after digestion with the appropriate restriction enzyme (EcoRI for the 5’ targeted region).
(TIF)

Figure S2 ***Mazr*^*F/F*^*Vav-iCre* mice display a normal development and growth phenotype.** (A) Diagram shows the number of *Mazr* ^*F/F*^ and *Mazr* ^*F/F*^ *Vav-iCre* offspring (total number is 229).
(B) Representative picture showing eight weeks old male *Mazr* ^*F/F*^ and *Mazr* ^*F/F*^ *Vav-iCre* littermates. Horizontal bar indicates 1 cm.
(TIF)

Figure S3 **PCR strategy to determine *Mazr* deletion efficiency.** (A) PCR genotyping of DNA extracted from *Mazr* ^F/F^ and *Mazr* ^*F/F*^ *Vav-iCre* BM (left) and BM-derived mast cells (BMMCs, right).
(B) *Mazr* ^F/F^ and *Mazr* ^*F/F*^ *Vav-iCre* BM cells were mixed at the indicated ratio and PCR was performed to detect the deleted *Mazr* alleles. The detection strategy of F (“floxed”) (PCR 1+2) and ∆ (PCR 1+3) alleles and the approximate location of the PCR primers is shown in Figure S1B. The size of the PCR fragments are 341 bp (for F) 304 bp (for Δ). Data shown are representative of 2 independent experiments.
(TIF)

Figure S4 **Gating strategy to determine the percentages of peritoneal mast cells.** (A) Representative flow cytometry analysis of peritoneal lavage cells of wild-type (*Mazr^F/+^Rosa26^+/EYFP^Mcpt5Cre*) and mast cell-specific MAZR-null (*Mazr^F/F^Rosa26^+/EYFP^Mcpt5Cre*) mice. Left panel shows side versus forward scatter plot and the gating region for cells that include peritoneal mast cells. The FSC/EYFP plot shows the gating strategy for EYFP^+^ cells. The dot plots in the right panel indicates the percentage of MC (defined as c-kit^+^FcεRI^+^) among the EYFP^+^ population.
(B) Summary of the percentage of c-kit^+^FcεRI^+^ mast cells within the EYFP^+^ population of peritoneal lavages of wild-type (*Mazr^F/+^Rosa26^+/EYFP^Mcpt5Cre*) and mast cell-specific MAZR-null (*Mazr^F/F^Rosa26^+/EYFP^Mcpt5Cre*) mice (n=8 and 10, respectively). Mean ± SEM is shown.
(TIF)

Table S1 **Primers for qRTPCR analysis.** List of primers used for qRTPCR analysis. *Hprt* primers were taken from Ref [7].. The rest of the primers were designed with the use of Primer3web version 4.0.0 [29].
(DOCX)

Table S2 **Genotyping primers.** List of primers used for genotyping of mice. The primers were taken from the following references: *Mazr* [8], *Vav-iCre* [18], EYFP and *Mcpt5Cre* [19].
(DOCX)

Table S3 **Up- and down-regulated genes in the absence of MAZR.** List of all genes that are differentially expressed (≥2 fold-change (FC), P≤0.1) between IgE-primed *Mazr* ^F/F^ and *Mazr* ^*F/F*^ *Vav-iCre* BMMCs. Gene expression profiles were determined using Agilent arrays and GeneSpring software as described in materials and methods. Only those probes with assigned gene names are listed (long intergenic non-coding (linc) RNAs are excluded from the list).
(DOCX)

Table S4 **GO classification of up-and down-regulated genes.** Probe numbers or the genes identified by Agilent arrays as dysregulated in the absence of MAZR were run through The Database for Annotation, Visualization and Integrated Discovery (DAVID) v6.7 Bioinformatics Database (http://david.abcc.ncifcrf.gov; [22,23]) Default (medium) setting of analysis was used to identify clusters of genes based on either biological process they are implicated in or their molecular function (only cluster containing 3 or more genes are indicated). Each gene can possibly fall into more than one cluster. The enrichment scores of the GO categories are indicated. Of the 103 genes that were up-regulated in the absence of MAZR, 94 were accepted by the database for analysis. For biological process clustering 24 genes were not clustered, whereas for molecular function 6 genes were not clustered. Of the 25 genes that were down-regulated in the absence of MAZR, 24 were accepted by the database for analysis. For both biological process and molecular process clustering 1 gene was not clustered.
(DOCX)
