# Supplementary figures and images for: The Transcription Factor MAZR Preferentially Acts as a Transcriptional Repressor in Mast Cells and Plays a Minor Role in the Regulation of Effector Functions in Response to FcεRI Stimulation
Source: PLoS One. 2013 Oct 17;8(10):e77677. doi: 10.1371/journal.pone.0077677 (PMC3804165; doi:10.1371/journal.pone.0077677)

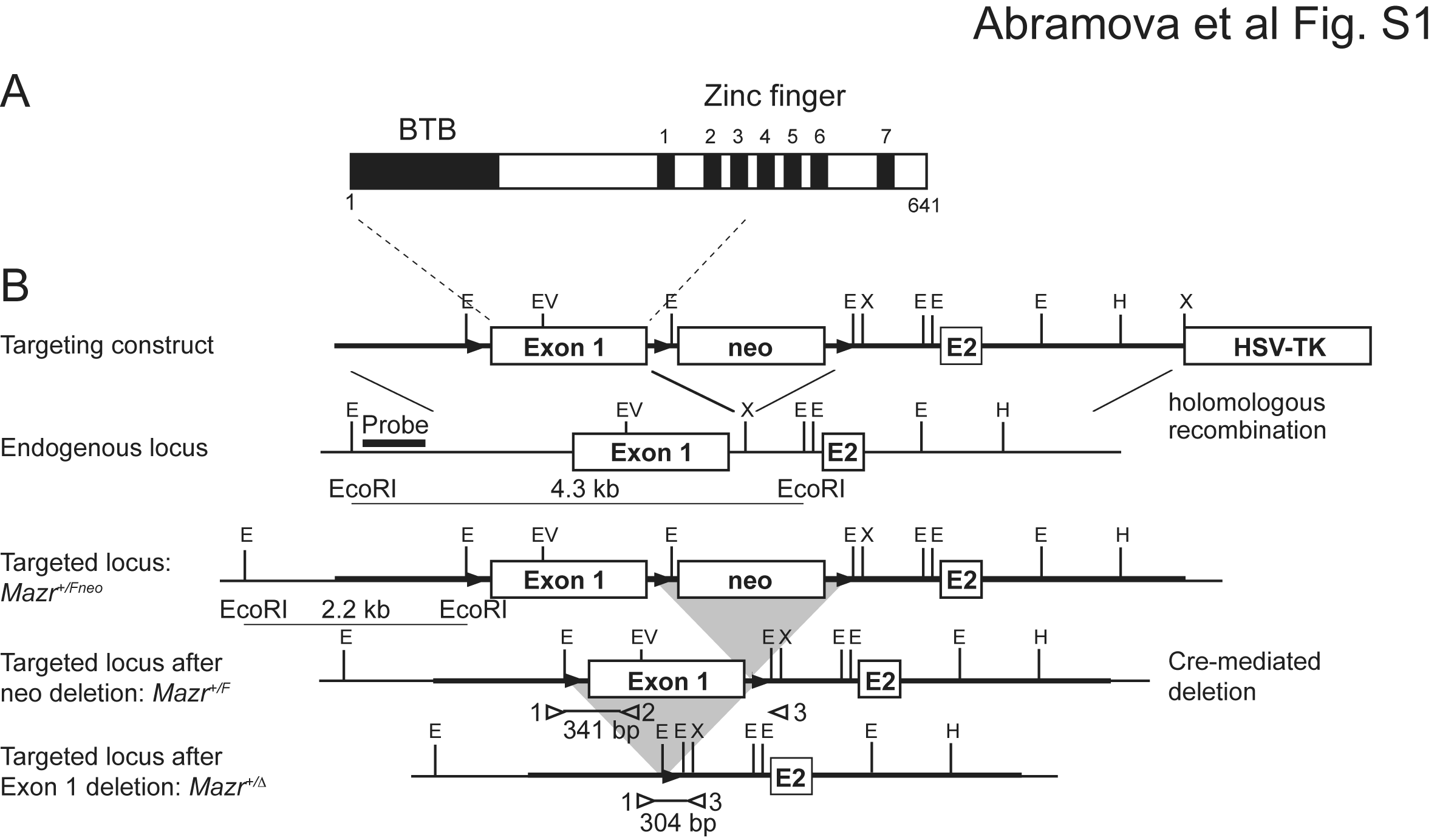

Supplement: Figure S1 — Generation of a conditional Mazr allele. (A) Schematic map of MAZR (641 aa) showing the N-terminal BTB domain and the 7 zinc fingers at the C-terminus. (B) Targeting strategy for Mazr (as previously shown in [8]). Schematic map of the targeting construct (top panel), the endogenous Mazr gene locus (middle panel), the targeted Mazr gene locus after homologous recombination (Mazr +/Fneo; upper bottom panel), and the targeted locus after the neomycin cassette deletion (Mazr +/F; middle bottom panel) and iCre recombinase-mediated deletion of exon 1 (Mazr +/Δ; lower bottom panel, respectively). All EcoRI (E), XhoI (X) and EcoRV (EV) restriction sites are shown. Open arrowheads (1, 2, and 3) indicate the location of the PCR primers used to detect deletion of exon 1. The horizontal thick black line (in the top and bottom panel) indicates the region of homology between the targeting construct and the endogenous locus. The thick bar (in the middle part) represents the 5' probe used for Southern-blotting. Horizontal bars with numbers (indicating the size in kb) show the expected genomic fragments after digestion with the appropriate restriction enzyme (EcoRI for the 5’ targeted region). (TIF) [file pone.0077677.s001.tif]

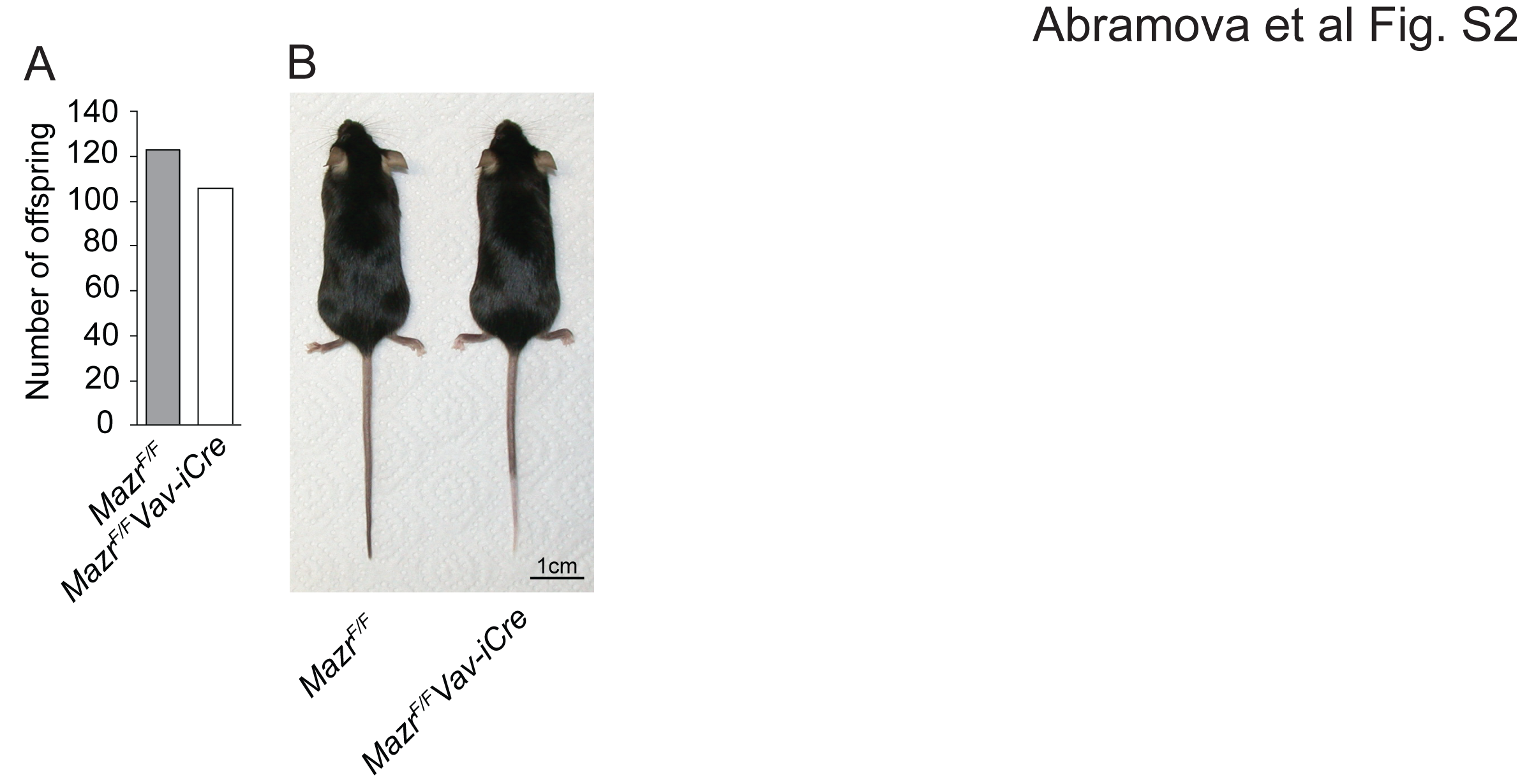

Supplement: Figure S2 — MazrF/FVav-iCre mice display a normal development and growth phenotype. (A) Diagram shows the number of Mazr F/F and Mazr F/F Vav-iCre offspring (total number is 229). (B) Representative picture showing eight weeks old male Mazr F/F and Mazr F/F Vav-iCre littermates. Horizontal bar indicates 1 cm. (TIF) [file pone.0077677.s002.tif]

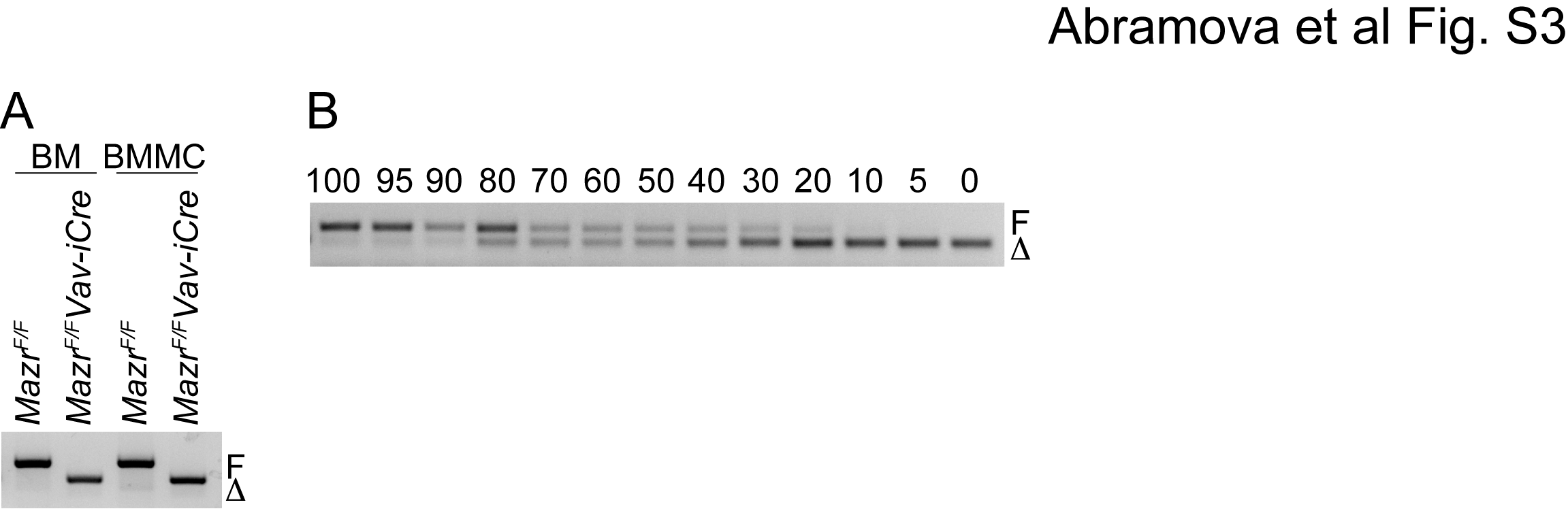

Supplement: Figure S3 — PCR strategy to determine Mazr deletion efficiency. (A) PCR genotyping of DNA extracted from Mazr F/F and Mazr F/F Vav-iCre BM (left) and BM-derived mast cells (BMMCs, right). (B) Mazr F/F and Mazr F/F Vav-iCre BM cells were mixed at the indicated ratio and PCR was performed to detect the deleted Mazr alleles. The detection strategy of F (“floxed”) (PCR 1+2) and ∆ (PCR 1+3) alleles and the approximate location of the PCR primers is shown in Figure S1B. The size of the PCR fragments are 341 bp (for F) 304 bp (for Δ). Data shown are representative of 2 independent experiments. (TIF) [file pone.0077677.s003.tif]

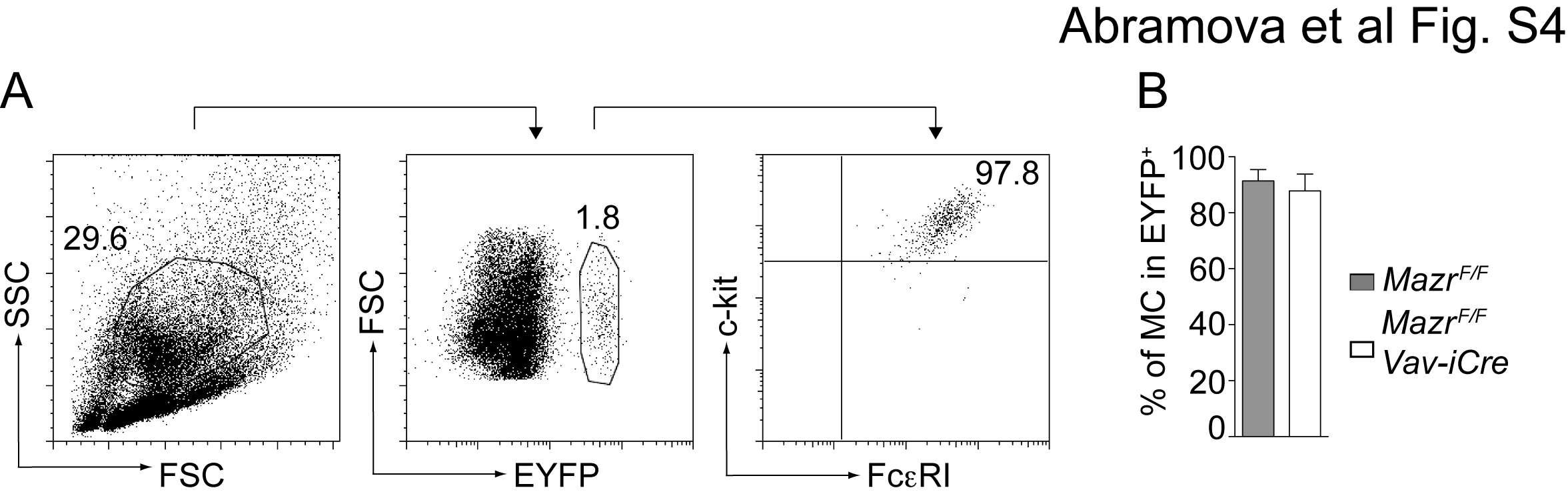

Supplement: Figure S4 — Gating strategy to determine the percentages of peritoneal mast cells. (A) Representative flow cytometry analysis of peritoneal lavage cells of wild-type (MazrF/+Rosa26+/EYFPMcpt5Cre) and mast cell-specific MAZR-null (MazrF/FRosa26+/EYFPMcpt5Cre) mice. Left panel shows side versus forward scatter plot and the gating region for cells that include peritoneal mast cells. The FSC/EYFP plot shows the gating strategy for EYFP+ cells. The dot plots in the right panel indicates the percentage of MC (defined as c-kit+FcεRI+) among the EYFP+ population. (B) Summary of the percentage of c-kit+FcεRI+ mast cells within the EYFP+ population of peritoneal lavages of wild-type (MazrF/+Rosa26+/EYFPMcpt5Cre) and mast cell-specific MAZR-null (MazrF/FRosa26+/EYFPMcpt5Cre) mice (n=8 and 10, respectively). Mean ± SEM is shown. (TIF) [file pone.0077677.s004.tif]
