# Supplementary material for: The Transcription Factor MAZR Preferentially Acts as a Transcriptional Repressor in Mast Cells and Plays a Minor Role in the Regulation of Effector Functions in Response to FcεRI Stimulation
Source: PLoS One. 2013 Oct 17;8(10):e77677. doi: 10.1371/journal.pone.0077677 (PMC3804165; doi:10.1371/journal.pone.0077677)
Supplement: Table S1 — Primers for qRTPCR analysis. List of primers used for qRTPCR analysis. Hprt primers were taken from Ref [7].. The rest of the primers were designed with the use of Primer3web version 4.0.0 [29]. (DOCX) [file pone.0077677.s005.docx]

**Table S1. Primers for qRTPCR analysis.**

| **Gene** | **Forward** (5’ - 3’) | **Reverse** (5’ - 3’) |
| --- | --- | --- |
| *Hprt* | ATTGTGGCCCTCTGTGTGCT | TTGCGCTCATCTTAGGCTTTG |
| *Mazr* | TGTGTGGTCTGCGGTTCAAG | GAGCATTTCTGGCCTTCTCG |
| *Cxcl10* | GTCCTAATTGCCCTTGGTCT | CGCACCTCCACATAGCTTAC |
| *Ccl5* | CAATCTTGCAGTCGTGTTTG | GATTTCTTGGGTTTGCTGTG |
| *Il18* | TGAAGGACACTTTCTTGCTTG | GCCTCGGGTATTCTGTTATG |
| *Cxcl12* | CAACGTCAAGCATCTGAAAA | TCCACTTTAATTTCGGGTCA |
| *Arhgef18* | CAAACTCAAAAACGGGCTAA | CTCGTTGGCTACCTCTCTCA |
| *Sel1l3* | CGCAGTAGCGAAGAATTACAC | TGGCCTCTAGCTCTCTGTTC |
| *Sva1* | ACACAATGAACCCCGTAACT | ATCCCAGAAGAAATCGCTAA |
| *Ccr5* | AAGCTGCAAAAAGCTGAAGA | TAAAGCAAACACAGCATGGA |
| *Mpped2* | TTCAGTCCCTCCTGACAAAC | GAACCCAGTCTCGAAAACCT |
