# Supplementary material for: The Transcription Factor MAZR Preferentially Acts as a Transcriptional Repressor in Mast Cells and Plays a Minor Role in the Regulation of Effector Functions in Response to FcεRI Stimulation
Source: PLoS One. 2013 Oct 17;8(10):e77677. doi: 10.1371/journal.pone.0077677 (PMC3804165; doi:10.1371/journal.pone.0077677)
Supplement: Table S2 — Genotyping primers. List of primers used for genotyping of mice. The primers were taken from the following references: Mazr [8], Vav-iCre [18], EYFP and Mcpt5Cre [19]. (DOCX) [file pone.0077677.s006.docx]

**Supplementary Table 2. Genotyping primers.**

| **Genotyping primers** | |
| --- | --- |
| *Mazr* | GGGTCTAGCCCTTTTTATTAGAGC |
|  | GAAGCTCTCGTCGCCTACTC |
|  | GTTATGTTCTATTAAGGTCCAGTGACC |
| *Vav-iCre* | CCGAGGGGCCAAGTGAGAGG |
|  | GGAGGGCAGGCAGGTTTTGGTG |
| *EYFP* | CCAAAGTCGCTCTGAGTTGTTATC |
|  | GCGAAGAGTTTGTCCTCAACC |
|  | GGAGCGGGAGAAATGGATATG |
| *Mcpt5Cre* | ACAGTGGTATTCCCGGGGAGTGT |
|  | GTCAGTGCGTTCAAAGGCCA |
