# Supplementary material for: The Transcription Factor MAZR Preferentially Acts as a Transcriptional Repressor in Mast Cells and Plays a Minor Role in the Regulation of Effector Functions in Response to FcεRI Stimulation
Source: PLoS One. 2013 Oct 17;8(10):e77677. doi: 10.1371/journal.pone.0077677 (PMC3804165; doi:10.1371/journal.pone.0077677)
Supplement: Table S3 — Up- and down-regulated genes in the absence of MAZR. List of all genes that are differentially expressed (≥2 fold-change (FC), P≤0.1) between IgE-primed Mazr F/F and Mazr F/F Vav-iCre BMMCs. Gene expression profiles were determined using Agilent arrays and GeneSpring software as described in materials and methods. Only those probes with assigned gene names are listed (long intergenic non-coding (linc) RNAs are excluded from the list). (DOCX) [file pone.0077677.s007.docx]

**Table S3. Up-regulated genes in the absence of MAZR.**

| **#** | **Probe number** | **FC** | **Gene name** |
| --- | --- | --- | --- |
| 1 | A_55_P2136184 | 22,57 | *Sva* |
| 2 | A_55_P2129771 | 13,06 | *Prrt1* |
| 3 | A_51_P291078 | 10,20 | *Sel1l3* |
| 4 | A_52_P638459 | 8,50 | *Ccl5* |
| 5 | A_51_P172502 | 8,34 | *Cxcl12* |
| 6 | A_51_P327751 | 7,16 | *Ifit1* |
| 7 | A_55_P2016462 | 6,95 | *Cxcl10* |
| 8 | A_52_P346987 | 6,32 | *Hoxc9* |
| 9 | A_55_P1983588 | 6,19 | *Pmepa1* |
| 10 | A_55_P1960857 | 6,12 | *Sorbs2* |
| 11 | A_55_P2384593 | 5,73 | *Gpc6* |
| 12 | A_51_P515120 | 5,70 | *Hs3st3a1* |
| 13 | A_55_P2205226 | 5,56 | *6430519N07Rik* |
| 14 | A_51_P144957 | 5,39 | *Tram1l1* |
| 15 | A_51_P282227 | 5,07 | *As3mt* |
| 16 | A_55_P1998687 | 5,05 | *Gm10487* |
| 17 | A_52_P356698 | 4,97 | *Shox2* |
| 18 | A_51_P163953 | 4,57 | *Nsg2* |
| 19 | A_55_P1982186 | 4,55 | *Sgsm1* |
| 20 | A_55_P2040170 | 4,46 | *Pmp22* |
| 21 | A_51_P382912 | 4,40 | *Slc2a10* |
| 22 | A_55_P2095213 | 4,19 | *Dusp22* |
| 23 | A_52_P276955 | 4,16 | *Epha3* |
| 24 | A_51_P384318 | 4,16 | *C1ra* |
| 25 | A_55_P2380806 | 3,97 | *Gm2115* |
| 26 | A_52_P363216 | 3,72 | *Gcnt2* |
| 27 | A_55_P2130090 | 3,68 | *Cnn3* |
| 28 | A_52_P69020 | 3,65 | *Slc24a5* |
| 29 | A_52_P322421 | 3,64 | *Mpzl2* |
| 30 | A_55_P2074688 | 3,59 | *Hoxc4* |
| 31 | A_66_P140452 | 3,52 | *Mtap1a* |
| 32 | A_55_P1958160 | 3,44 | *Sgce* |
| 33 | A_55_P2156731 | 3,43 | *H2-Eb1* |
| 34 | A_66_P105046 | 3,42 | *Il18* |
| 35 | A_51_P155085 | 3,39 | *Dennd2a* |
| 36 | A_51_P438619 | 3,37 | *Sobp* |
| 37 | A_51_P450169 | 3,36 | *Dnajb13* |
| 38 | A_52_P578732 | 3,25 | *Ccr5* |
| 39 | A_51_P102421 | 3,17 | *Clcf1* |
| 40 | A_66_P139094 | 3,17 | *Mrgpra9* |
| 41 | A_51_P327983 | 3,15 | *4833427G06Rik* |
| **#** | **Probe number** | **FC** | **Gene name** |
| 42 | A_55_P2103596 | 3,10 | *Slc30a4* |
| 43 | A_52_P1093529 | 3,10 | *Pik3r5* |
| 44 | A_52_P536022 | 3,06 | *Rasgrf1* |
| 45 | A_55_P2090330 | 3,05 | *Kcnmb4* |
| 46 | A_51_P330044 | 3,03 | *Cyp2j9* |
| 47 | A_52_P343306 | 2,99 | *H2-Aa* |
| 48 | A_55_P2039284 | 2,98 | *Hspb1* |
| 49 | A_51_P491350 | 2,98 | *Col4a2* |
| 50 | A_51_P138895 | 2,95 | *Ccdc102a* |
| 51 | A_55_P2292046 | 2,94 | *D230018H15Rik* |
| 52 | A_55_P2000127 | 2,86 | *Tgfb1i1* |
| 53 | A_55_P2282331 | 2,79 | *4930484H19Rik* |
| 54 | A_51_P393654 | 2,79 | *Fam171b* |
| 55 | A_51_P187262 | 2,79 | *Mmp25* |
| 56 | A_55_P2096797 | 2,78 | *Nlrp1b* |
| 57 | A_51_P303424 | 2,76 | *Itgax* |
| 58 | A_55_P2143070 | 2,75 | *Ass1* |
| 59 | A_52_P639229 | 2,74 | *Fgd4* |
| 60 | A_55_P2106180 | 2,67 | *Dnahc2* |
| 61 | A_52_P585652 | 2,67 | *Fndc3b* |
| 62 | A_55_P1982454 | 2,64 | *Eps8* |
| 63 | A_55_P2038747 | 2,54 | *Ano1* |
| 64 | A_66_P128297 | 2,54 | *4930417O13Rik* |
| 65 | A_55_P1992749 | 2,52 | *Epb4,1l1* |
| 66 | A_55_P2031836 | 2,50 | *Gpr85* |
| 67 | A_55_P2050754 | 2,50 | *Cd209c* |
| 68 | A_55_P2065909 | 2,45 | *Macrod2* |
| 69 | A_52_P40832 | 2,45 | *Rab11fip4* |
| 70 | A_55_P2037817 | 2,44 | *Palmd* |
| 71 | A_55_P2140118 | 2,41 | *Qpct* |
| 72 | A_66_P100765 | 2,38 | *Dao* |
| 73 | A_51_P346575 | 2,37 | *Rhag* |
| 74 | A_55_P2096370 | 2,35 |  |
| 75 | A_55_P2334484 | 2,33 | *D9Ertd115e* |
| 76 | A_66_P100794 | 2,31 | *Magea5* |
| 77 | A_52_P480351 | 2,30 | *Loxl2* |
| 78 | A_55_P2059010 | 2,30 | *Rbp1* |
| 79 | A_55_P2097367 | 2,29 | *4933432B09Rik* |
| 80 | A_51_P207751 | 2,28 | *Guca1a* |
| 81 | A_55_P2096917 | 2,27 | *Mreg* |
| 82 | A_66_P140082 | 2,27 | *5031410I06Rik* |
| **#** | **Probe number** | **FC** | **Gene name** |
| 83 | A_52_P282741 | 2,25 | *Sdc3* |
| 84 | A_66_P105032 | 2,24 | *Gm13889* |
| 85 | A_52_P465946 | 2,24 | *Olfr275* |
| 86 | A_51_P314397 | 2,23 | *Crip2* |
| 87 | A_65_P10913 | 2,21 | *Tgfb2* |
| 88 | A_66_P124179 | 2,20 | *Atp6v0d2* |
| 89 | A_55_P2118717 | 2,20 | *Ninl* |
| 90 | A_51_P156274 | 2,19 | *Ltbr* |
| 91 | A_55_P2029711 | 2,15 | *Cdh2* |
| 92 | A_55_P2103320 | 2,15 | *Gm12508* |
| 93 | A_55_P2150003 | 2,15 | *Gnat3* |
| **#** | **Probe number** | **FC** | **Gene name** |
| 94 | A_55_P2019457 | 2,14 | *Eno2* |
| 95 | A_55_P2063312 | 2,13 | *Mgll* |
| 96 | A_55_P2078433 | 2,13 | *Mcoln2* |
| 97 | A_51_P362209 | 2,11 | *Commd10* |
| 98 | A_55_P1976097 | 2,11 | *Fads3* |
| 99 | A_52_P100926 | 2,10 | *Il1a* |
| 100 | A_55_P2015541 | 2,04 | *Hif3a* |
| 101 | A_51_P303620 | 2,03 | *Whrn* |
| 102 | A_55_P1962305 | 2,00 | *Plac8* |
| 103 | A_51_P292008 | 2,00 | *Gpx3* |

**Table S3 (continued). Down-regulated in the absence of MAZR.**

| **#** | **Probe number** | **FC** | **Gene name** |
| --- | --- | --- | --- |
| 1 | A_52_P408025 | 6,55 | *Mpped2* |
| 2 | A_52_P476431 | 6,07 | *Evc* |
| 3 | A_55_P2079659 | 4,82 | *Dnase2b* |
| 4 | A_52_P441974 | 4,61 | *Evc2* |
| 5 | A_55_P2033917 | 3,91 | *Fut10* |
| 6 | A_51_P197137 | 3,71 | *Olfr370* |
| 7 | A_55_P2172096 | 3,64 | *Mc1r* |
| 8 | A_55_P2176953 | 3,25 | *Usp51* |
| 9 | A_55_P2039379 | 3,09 | *Slc27a6* |
| 10 | A_55_P1980321 | 2,95 | *Pcbp3* |
| 11 | A_52_P533040 | 2,92 | *Olfr805* |
| 12 | A_55_P2064862 | 2,90 | *Ica1* |
| 13 | A_51_P116906 | 2,71 | *Rapgef3* |
| 14 | A_55_P2278775 | 2,61 | *9130016M20Rik* |
| 15 | A_51_P394735 | 2,61 | *Arhgef18* |
| 16 | A_55_P2184339 | 2,46 | *Mdga1* |
| 17 | A_51_P302358 | 2,46 | *Ltb* |
| 18 | A_55_P1988398 | 2,39 |  |
| 19 | A_55_P2051716 | 2,36 | *Tbx1* |
| 20 | A_51_P260850 | 2,30 | *Cntnap2* |
| 21 | A_55_P2060349 | 2,24 | *Lzts2* |
| 22 | A_55_P2121980 | 2,19 | *Plcb4* |
| 23 | A_55_P2231342 | 2,10 | *A030010E16Rik* |
| 24 | A_51_P261379 | 2,09 | *Spats2* |
| 25 | A_52_P231232 | 2,05 | *Nanos1* |
