# Supplementary material for: The Transcription Factor MAZR Preferentially Acts as a Transcriptional Repressor in Mast Cells and Plays a Minor Role in the Regulation of Effector Functions in Response to FcεRI Stimulation
Source: PLoS One. 2013 Oct 17;8(10):e77677. doi: 10.1371/journal.pone.0077677 (PMC3804165; doi:10.1371/journal.pone.0077677)
Supplement: Table S4 — GO classification of up-and down-regulated genes. Probe numbers or the genes identified by Agilent arrays as dysregulated in the absence of MAZR were run through The Database for Annotation, Visualization and Integrated Discovery (DAVID) v6.7 Bioinformatics Database (http://david.abcc.ncifcrf.gov; [22,23]) Default (medium) setting of analysis was used to identify clusters of genes based on either biological process they are implicated in or their molecular function (only cluster containing 3 or more genes are indicated). Each gene can possibly fall into more than one cluster. The enrichment scores of the GO categories are indicated. Of the 103 genes that were up-regulated in the absence of MAZR, 94 were accepted by the database for analysis. For biological process clustering 24 genes were not clustered, whereas for molecular function 6 genes were not clustered. Of the 25 genes that were down-regulated in the absence of MAZR, 24 were accepted by the database for analysis. For both biological process and molecular process clustering 1 gene was not clustered. (DOCX) [file pone.0077677.s008.docx]

**Table S4. GO classification of up-and down-regulated genes.**

| **Cluster** | **Biological process** | **Enrichment score** | **Number of genes** |
| --- | --- | --- | --- |
| 1 | Locomotory behavior/Chemotaxis | 1.90 | 7 |
| 2 | Inflammatory/Immune response | 1.87 | 11 |
| 3 | Positive regulation of immune response | 1.27 | 6 |
| 4 | Adaptive immune response | 1.16 | 4 |
| 5 | Cell proliferation/Neuron development | 0.88 | 7 |
| 6 | Hemopoiesis and immune system development | 0.67 | 4 |
| 7 | Blood vessel development and cell migration | 0.64 | 4 |
| 8 | Ion transport and cellular homeostasis | 0.62 | 12 |
| 9 | Cytoskeleton organization | 0.56 | 3 |
| 10 | Cell activation | 0.54 | 3 |
| 11 | Embryonic development and transcription regulation | 0.51 | 7 |
| 12 | Sensory perception | 0.49 | 7 |
| 13 | Phospho- related metabolic processes | 0.21 | 5 |
| 14 | Regulation of apoptosis | 0.11 | 3 |
| 15 | Protein localization and transport | 0.07 | 3 |
| **Cluster** | **Molecular function** | **Enrichment score** | **Number of genes** |
| 1 | Cytokine/Chemokine activity or receptor binding | 2.20 | 6 |
| 2 | Ion binding | 0.78 | 21 |
| 3 | GTPase regulator activity | 0.39 | 3 |
| 4 | Channel or passive transporter activity | 0.31 | 3 |
| 5 | Transcription and DNA binding activity | 0.08 | 4 |
| 6 | ATP/Nucleotide/Nucleoside activity | 0.01 | 5 |

**GO classification of down-regulated genes**

| **Cluster** | **Biological process** | **Enrichment score** | **Number of genes** |
| --- | --- | --- | --- |
| 1 | Sensory perception/Neurological system process/ G-protein coupled receptor signaling | 0.39 | 5 |
| **Cluster** | **Molecular function** | **Enrichment score** | **Number of genes** |
| 1 | Ion binding | 0.07 | 4 |
